# A cerebro-cerebellar network for learning visuomotor associations

**DOI:** 10.1038/s41467-024-46281-0

**Published:** 2024-03-21

**Authors:** Naveen Sendhilnathan, Andreea C. Bostan, Peter L. Strick, Michael E. Goldberg

**Affiliations:** 1 Doctoral program in Neurobiology and Behavior, Columbia University, New York, NY USA; 2 Dept. of Neuroscience, Mahoney Center for Brain and Behavior Research, Zuckerman Mind, Brain, and Behavior Institute, Columbia University, New York, NY USA; 3 Department of Neurobiology, Systems Neuroscience Center, and Brain Institute, University of Pittsburgh, Pittsburgh, PA USA; 4 Kavli Institute for Brain Science, Columbia University, New York, NY USA; 5 Dept. of Neurology, Psychiatry, and Ophthalmology, Columbia University College of Physicians and Surgeons, New York, NY USA

**Keywords:** Cognitive control, Cerebellum

## Abstract

Consensus is rapidly building to support a role for the cerebellum beyond motor function, but its contributions to non-motor learning remain poorly understood. Here, we provide behavioral, anatomical and computational evidence to demonstrate a causal role for the primate posterior lateral cerebellum in learning new visuomotor associations. Reversible inactivation of the posterior lateral cerebellum of male monkeys impeded the learning of new visuomotor associations, but had no effect on movement parameters, or on well-practiced performance of the same task. Using retrograde transneuronal transport of rabies virus, we identified a distinct cerebro-cerebellar network linking Purkinje cells in the posterior lateral cerebellum with a region of the prefrontal cortex that is critical in learning visuomotor associations. Together, these results demonstrate a causal role for the primate posterior lateral cerebellum in non-motor, reinforcement learning.

## Abstract (web summary)

The extent of cerebellar contributions to non-motor learning remains unclear. Here, authors identify a cortico-cerebellar circuit in primates that plays a causal role in reinforcement-based learning of visuomotor associations.

## Introduction

Cerebellar circuits with the cerebral cortex in human and nonhuman primates are remarkably expansive^1–3^ and yet, for decades, the behavioral contributions of the cerebellum have been conceptualized almost exclusively within the realm of motor control^4^. The focus on cerebellar contributions to movement is unsurprising, given the well-established neuroanatomical circuits that allow the cerebellum to influence the motor cortex^5,6^, as well as converging evidence for cerebellar involvement in motor function from patient, imaging, electrophysiological, behavioral, and computational modeling studies^7–17^.

Spurred by the Leiners’ original observation^18^ that the evolutionary expansion of the cerebellar hemispheres parallels that of the prefrontal cortex, consensus has been gathering to support a cerebellar role beyond motor control. Anatomical tracing studies have shown that the cerebellum sends outputs not only to motor, but also to non-motor areas in the cerebral cortex^19–25^. Clinical evaluation of patients with cerebellar pathology and neuroimaging studies provide further evidence for cerebellar involvement in a variety of non-motor processes, including executive function, working memory, timing, language, and emotion^16,20,26–34^. However, given that the cerebellar cortex receives both motor and non-motor information^35,36^ crucial aspects of cerebellar contributions to non-motor functions remain unknown, including: (1) the cerebellum’s causal necessity for non-motor learning, (2) the computations that the cerebellum contributes to non-motor functions and whether they are independent from motor information, (3) the cerebral cortical regions that engage the cerebellum for non-motor learning, and (4) the cerebellar cortical regions that can affect non-motor learning at the level of the prefrontal cortex.

We previously showed that when a monkey learns a new visuomotor association, the simple spikes (SS) of Purkinje cells in Crus I and Crus II become responsive to the outcome of the prior trial, with roughly half of the neurons signaling that the monkey received a reward on the prior trial (correct-preferring cells, cP cells), and half signaling that the monkey failed to receive a reward (wrong-preferring cells, wP cells)^35^. The difference in the activity of these two types of neurons provides an error signal, which approaches zero as the monkey learns the association. When the monkey performs an overtrained visuomotor association task, the neurons are not sensitive to reward or errors. The complex spikes of the same neurons encode a signal predicting the start of a new trial, probability of failure in the current trial, but not the trial-by-trial error, signals which we interpreted as arousal or motivational signals^37^.

Here, we show that reversible inactivation of Crus I and II impaired learning new visuomotor associations, but not performance of the overtrained visuomotor association. Finally, using retrograde transneuronal transport of rabies virus, we determined that the same region of cerebellar cortex is interconnected with an area of the prefrontal cortex, the PrePMd, that has an established role in learning visuomotor associations^38^. This combination of behavioral, anatomical, and computational analyses establishes that the posterior-lateral cerebellum is critical for non-motor, reinforcement-based learning in nonhuman primates.

## Results

### Lateral-posterior cerebellar inactivation interfered with learning of novel visuomotor associations

**Fig. 1:**
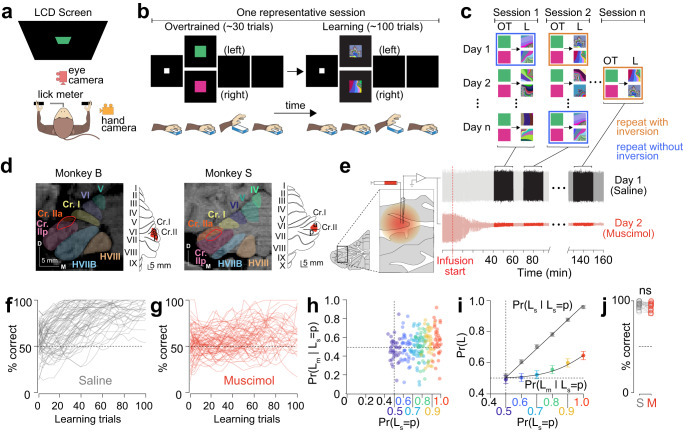
Lateral-posterior cerebellar inactivation caused an impairment in visuomotor association learning. **a** Schematic of the experimental setup. **b** Schematic of the two-alternative forced-choice discrimination task showing a representative session with ~30 trials of overtrained task (left) and ~100 trials of learning task (right). **c** Trial session organization. On a given experimental day, monkeys performed 3–10 sessions; Each session began with ~30 trials of overtrained symbols (green and pink squares) followed by a randomly chosen symbol pair from the library (Fig. S1a) presented until they learned them (~100 trails). Sessions marked with blue rectangles indicate repeated presentations of the same symbol pairs with same visuomotor associations as the last time the symbols were presented. Sessions marked with orange rectangles indicate repeated presentations of the same symbol pairs but with inverted visuomotor association (see “Methods”). We never repeated the same symbol pair with the same association on a given day. **d** For each monkey (B, left and S, right), the left panel shows a T1-weighted MRI image of the cerebellar hemisphere with muscimol infusion location (red outline) in the coronal plane. Lobules of interest are outlined in different colors. The right panel shows a flattened map reconstruction of the cerebellar cortex surface, with the infusion location marked in red. Scale bar 5 mm. Cr crus, D dorsal, M medial. **e** Continuous voltage traces from example saline (top) and muscimol infusion days (bottom) from the lateral-posterior cerebellum. **f** Behavioral performance for both monkeys for each session during novel learning task during control-saline condition. **g** Same as **f** but for lateral-posterior cerebellar inactivation condition. **h** Probability of learning in lateral-posterior cerebellar inactivation condition given the probability of learning in the control condition, $\Pr ({L}_{m}|{L}_{s}=p)$, plotted againts the probability of learning in the control condition $\Pr ({L}_{s}=p)$. Each marker is a session. The marker colors correspond to the colored values in the abscissa. m, muscimol and s, saline. **i** Quantitation from h. Data are shown as mean ± SEM. **j** Percent correct for each symbol pair during overtrained task in saline (S; gray) and muscimol (M; red) conditions. *P* = 0.32, n = 25, Mann–Whitney *U* test. Data are shown as mean ± SEM. Source data are provided as a Source Data file.

We trained two monkeys to associate arbitrary visual symbols with left and right-hand bar release to earn an immediate liquid reward (Fig. 1a, b, “Methods”). We created a library of 16 pairs of novel fractal symbols with varying levels of fractal image complexity and varying levels of similarity between the symbols in each pair (Fig S1a). On each recording session, after the monkeys performed ~30 trials with familiar, overtrained symbols (OT), we presented them with one of the novel symbol pairs randomly chosen from the library and tracked their learning. Since different symbol pairs in the library had different levels of complexities and similarities (Fig S1b–d), the number of trials each monkey took to learn the association differed among the different symbol pairs. To get a robust estimate of the difficulty of each symbol pair, we recorded the monkeys’ performance for each symbol pair (Monkey B- 16 pairs, Monkey S- 9 pairs) at least three times, spread over several weeks, repeatedly presented in random order (Fig. 1c; ”Methods”). The acquisition-difficulty levels (the trial when they reached 90% correct for each repetition of each symbol pair) were not significantly different among the repeated presentations of the same symbol pair (Fig S1b; monkey B: *P* = 0.46; monkey S: *P* = 0.63, ANOVA) suggesting that the monkeys forgot their prior experience with a given symbol pair during each additional re-learning.

To determine the effect of inactivation of Crus I and II on visuomotor association learning, we infused 4–10 μl of a 10 mg/ml solution of muscimol, a GABA_A_ agonist that hyperpolarizes cell bodies without affecting fibers of passage, into Crus I and II of the lateral-posterior cerebellum of two monkeys, in the same locations where we recorded task-dependent P cells^39^ (“Methods”, Fig. 1d). Muscimol infusions into the cerebellar cortex result in depression of excitability of granule and P cells, consequent disinhibition of deep cerebellar nuclei activity and a depression of olivary excitability^40^. As a control, we infused saline on the days in between muscimol injections, in the same location as the muscimol injections. On a given day, we injected either saline or muscimol (never both) at the start of the experimental day, waited for 30–40 min after the injection, and recorded 3–10 sessions (where each session is ~30 trials of overtrained task followed by ~100 trials of novel condition; Fig. 1e).

During saline-control sessions, the monkeys typically learned the novel association in 50–70 trials (Fig. 1f). Cerebellar inactivation significantly impaired the learning for the same pairs of symbols used for the saline-control session (Figs. 1g and S1c). Since different symbol pairs had different acquisition-difficulty levels (Fig. S1b, d–e), we compared the learning behavior between the same pairs of symbols in saline and muscimol conditions (Methods). When the monkeys performed at 50% correct during control sessions, their performance was also close to 50% during inactivation sessions (Fig. 1h, i). However, as the performance of the monkeys improved during control sessions, the performance of the monkeys improved during inactivation sessions at a much slower rate. In particular, when the monkeys’ performance was about 100% during control sessions, their performance was only ~65% during inactivation session (Fig. 1h, i). Since this performance was significantly lower than that of the control condition but at the same time, above chance level this suggests that the inactivation of lateral-posterior cerebellum significantly impaired learning novel visuomotor associations but did not eliminate it entirely.

When the posterior-lateral cerebellum was inactivated, the monkeys had difficulty learning even the association that had the least acquisition difficulty (Fig. S1d–g). However, this was not because they developed a choice bias (Fig. S2a). Instead, it may have been the case that their learning strategy was affected (Fig. S2b, c). In the saline-control sessions, the monkeys adopted a win-stay lose-switch strategy near the beginning of learning and the percentage of trials in which they used this strategy increased throughout the session, but in the muscimol sessions they adopted this strategy much later (Fig. S2b). We previously modeled the learning process using a drift-diffusion reinforcement learning model. This model used a drift-diffusion process to capture the decision process that resulted in which choice of the hand to release, and it used the reinforcement learning model to capture the error signal from P-cell simple spike activity^39^. Here we modeled the muscimol effect by replacing the reinforcement learning error signal with Gaussian white noise (Fig S3, “Methods”, Supplementary Note 1).

On a few additional experimental sessions, we used an error-correction strategy: repeating the same symbol on the next trial when the monkey made an error until the monkey got it right (“Methods”). This significantly improved the monkeys’ rate of learning under saline-control session as expected (*P* = 0.0029, paired *t*-test) but not during cerebellar inactivation (*P* = 0.11; paired *t*-test) for the same number of trials, for the same symbol pairs (Fig. S4).

Although inactivation of the posterior-lateral cerebellar cortex impaired learning new associations, the monkeys continued to perform the overtrained task at close to 100% accuracy, not different from their performance during the saline-control condition (Monkey B: *P* = 0.21; Monkey S: *P* = 0.50; Mann–Whitney *U* test; Fig. 1j). This implies that although the posterior-lateral cerebellar cortex is critical for learning, consolidation resides elsewhere (Supplementary Note 2). We verified that the performance modulations across sessions, within a day, did not confound our observation (Fig. S5). More importantly, the monkeys performed the overtrained task with an accuracy close to 100%, between the different learning task sessions within an experimental day. Thus, the learning deficiency could not be due to any effect of the muscimol on the monkey’s ability to perform the components of the task that did not require learning.

### Lateral-posterior cerebellar inactivation did not affect gross motor kinematics

**Fig. 2:**
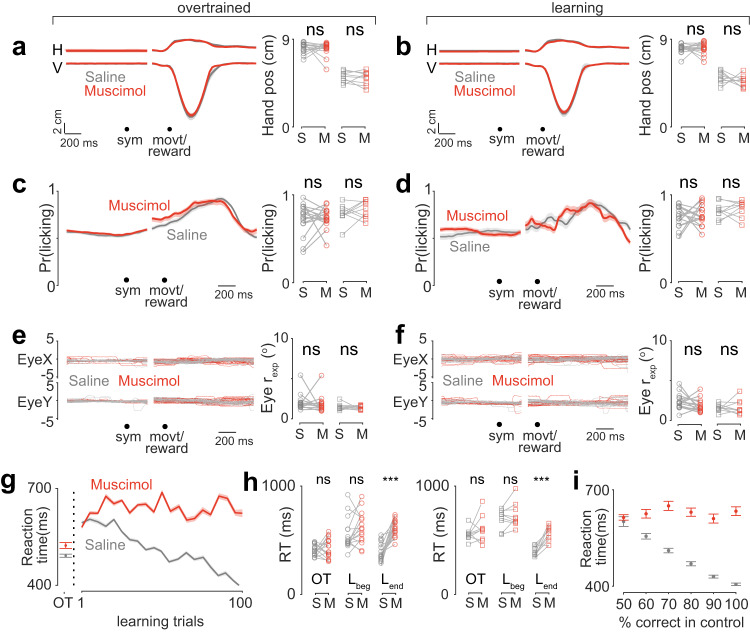
Lateral-posterior cerebellar inactivation did not cause any deficit in motor kinematics during visuomotor association learning. **a** Left: Mean horizontal (H) and vertical (V) hand positions across sessions for monkey B, during overtrained for control-saline (gray) and muscimol-inactivation (red) conditions. Right: Quantitation of the amplitude of hand movement for monkey B (left; circle markers; *P* = 0.73, *n* = 16, Mann–Whitney *U* test) and monkey S (right, square markers; *P* = 0.53; *n* = 9, paired *t*-test). S: saline, M: muscimol. **b** Same as **a**; but during learning. Right panel, monkey B (left; circle markers; *P* = 0.87, *n* = 16, paired t-test) and monkey S (right, square markers; *P* = 0.32; *n* = 9, paired *t*-test). **c** Left: Mean licking activity across sessions and monkeys during overtrained for control-saline (gray) and muscimol-inactivation (red) conditions. Right: Quantitation for monkey B (left; circle markers; *P* = 0.76, *n* = 16, paired t-test) and monkey S (right, square markers; *P* = 0.99; *n* = 9, paired *t*-test). **d** Same as **c**; but during learning. Right panel, monkey B (left; circle markers; *P* = 0.44, *n* = 16, paired t-test) and monkey S (right, square markers; *P* = 0.42; *n* = 9, paired t-test). **e** Left: X and Y raw eye-movement positions, from a representative session during overtrained for control-saline (gray) and muscimol-inactivation (red) conditions. Right: Quantitation of radius of visual exploration from all sessions (r_exp_; see “Methods”; Fig S6e–h) for monkey B (left; circle markers; *P* = 0.23, *n* = 16, Mann-Whitney *U* test) and monkey S (right, square markers; *P* = 0.73; *n* = 9, paired t-test). **f** Same as **e**; but during learning. Right panel, monkey B (left; circle markers; *P* = 0.11, *n* = 16, Mann–Whitney *U* test) and monkey S (right, square markers; *P* = 0.66; *n* = 9, Mann–Whitney *U* test). **g** Mean reaction time (RT) profile during overtrained and learning across sessions and monkeys for control-saline (gray) and inactivation (red) conditions. **h** Quantitation from G for two monkeys separately. *L*_beg_: beginning of learning and *L*_end_: end of learning. Left: Monkey B, OT: *P* = 0.48; paired *t*-test; *L*_beg_: *P* = 0.10; paired *t*-test; *L*_end_: *** *P* = 3.1909e-04 paired *t*-test; *n* = 16. Right: Monkey S, OT: *P* = 0.30; paired *t*-test; *L*_beg_: *P* = 0.28; paired *t*-test; ***L_end_: *P* = 0.0021 paired t-test, *n* = 9. **i** Reaction time during control (gray) and inactivation (red) conditions for different performances during control condition. Data are shown as mean ± SEM. Source data are provided as a Source Data file.

Although inactivation of the posterior-lateral cerebellum prevented the monkeys from learning new visuomotor associations, it did not affect the gross motor kinematics of hand movement: In both control and inactivation conditions, the monkeys performed the task with well-stereotyped hand movements in the overtrained task (Figs. 2a, S6a; Monkey B: *P* = 0.73; Mann–Whitney *U* test; Monkey S: *P* = 0.53; paired *t*-test) and during learning (Figs. 2b, S6b; Monkey B: *P* = 0.87; paired t-test; Monkey S: *P* = 0.32; paired *t*-test). It should be noted that in any case, the exact kinematics of the hand movement made by the monkeys did not affect reward probability and was task irrelevant (“Methods”). The licking behavior was also not different between the control and inactivation conditions for the overtrained tasks (Figs. 2c, S6c; Monkey B: *P* = 0.76; paired *t*-test; Monkey S: *P* = 0.99; paired t-test) or during learning (Figs. 2d, S6d; Monkey B: *P* = 0.44; paired *t*-test; Monkey S: *P* = 0.42; paired *t*-test). Although monkeys generally tended to look around the screen more during learning than during the overtrained tasks, there were no systematic differences in their eye movements between the control and inactivation conditions during the overtrained tasks (Figs. 2e, S6g; Monkey B: *P* = 0.23; Mann–Whitney *U* test; Monkey S: *P* = 0.73; paired *t*-test) or during learning (Figs. 2f, S6h; Monkey B: *P* = 0.11; Mann–Whitney *U* test; Monkey S: *P* = 0.66; Mann–Whitney *U* test). Because the symbol only appeared for 100 ms, half the usual saccadic reaction time of monkeys^41^ the cerebellar deficit in learning could not have been due to a failure in saccade-driven visual search as the monkeys sought to find features which distinguished between the two symbols (Fig S6i–k). Additionally, under cerebellar inactivation, the motor kinematics for correct and wrong trials were not different (Fig. S7). In a few additional experiments, we switched the manipulanda from bars to dowels, which required the monkeys to make entirely different hand movement to report the same choices (“Methods”). Posterior-lateral cerebellar inactivation had no effect on the kinematics of the changed movement (Fig. S8).

However, under inactivation of the posterior-lateral cerebellum, the reaction time did not decrease during learning, in keeping with the monkey’s failure to learn the symbol associations (Monkey B: *P* = 0.001 paired *t*-test; Monkey S: P = 0.01 paired *t*-test; Fig. 2g, h). This effect could be explained by the computational model described earlier (Fig. S3, Supplementary Note 1). Given that there were no changes in any aspect of the movements with which the monkeys signaled their decision, we inferred that inactivating the lateral-posterior cerebellum interfered with the monkeys’ ability to make a decision about the association, rather than their ability to report it.

### Anterior cerebellar inactivation did not interfere with learning of novel visuomotor associations

To test if the effect of muscimol inactivation were specific to the posterior-lateral cerebellum, we made similar muscimol injections into more anterior parts of the cerebellum in both monkeys (lobule V, an area that is interconnected with the primary motor cortex^19^ and likely involved in motor control^42^ (Figs. S9a and S10)). We again recorded the monkeys’ performance for a few randomly selected symbol pairs from the library (Fig. S1a; Monkey B- 5 pairs, Monkey S- 4 pairs). Inactivation of the anterior cerebellum had no effect on the monkey’s ability to learn new visuomotor associations (Monkey B: *P* = 0.71, paired *t*-test; Monkey S: *P* = 0.42, paired *t*-test; Fig. S9b, c) or to perform the overtrained task (Monkey B: *P* = 0.39, paired *t*-test; Monkey S: *P* = 0.38, paired *t*-test) suggesting that the anterior cerebellar inactivation did not significantly affect visuomotor association learning (Fig. S9d–h). One of the monkeys developed transient ataxia of the ipsilateral leg; the other developed nystagmus, showing that although anterior muscimol injection did not impair visuomotor learning, it impaired other behavioral functions.

### Identifying the cerebro-cerebellar network for learning new visuomotor associations

**Fig. 3:**
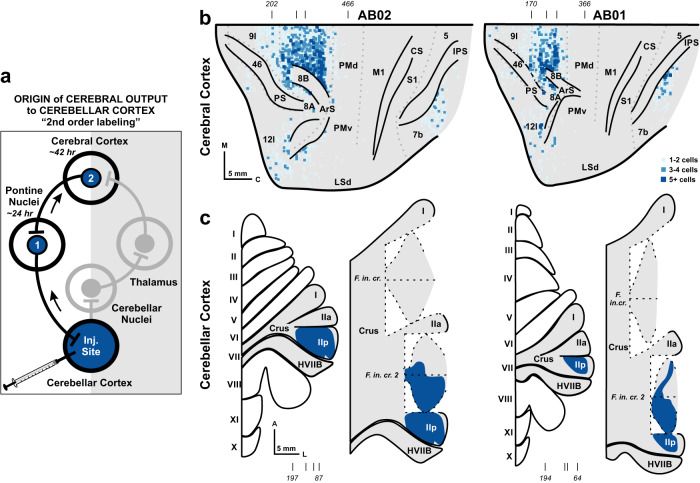
Origin of cerebral cortical output to the lateral-posterior cerebellum. **a** The schematic illustrates the experimental design and rabies virus transport in the cerebro-cerebellar circuits from injections into the cerebellar cortex. Regions containing rabies virus infected neurons are shown in blue. Virus is transported retrogradely from the injection site in cerebellar cortex to first-order neurons, such as neurons in the pontine nuclei. We estimate that this first stage of rabies transport takes approximately 24 h. At a survival time of 42 h, we observed neurons labeled by retrograde transneuronal transport of rabies virus to second-order neurons, including neurons in Layer V of the cerebral cortex. **b** The distribution of infected neurons following rabies virus injections in Crus II is illustrated on flattened surface maps of the lateral wall of the cerebral cortex in two cases (AB01 – Right; AB02 – Left). Colored squares indicate the number of infected neurons in successive 200-µm × 200-µm bins (color key). Estimate cytoarchitectonic boundaries are shown in light gray dotted lines. Tick marks (top) indicate level of sections depicted in Fig. S13. **c** Dark blue outlines indicate the extent of the cerebellar cortex injection sites on flattened maps of the cerebellar cortex in two cases (AB01 – Right; AB02 – Left). Shaded areas on the flattened surface maps are unfolded on the right side of each diagram. Tick marks (bottom) indicate level of sections depicted in Fig. S11. A anterior, ArS arcuate sulcus, C caudal, CS central sulcus, IPS intraparietal sulcus, l/L lateral, LSd dorsal lip of the lateral sulcus, M medial, M1 primary motor cortex, PMd dorsal premotor cortex, PMv ventral premotor cortex, PS principal sulcus, S1 primary sensory cortex.

To identify the network with which the neurons in the posterior-lateral cerebellum are connected, we injected the N2c strain of rabies virus into posterior portions of Crus II in cebus monkeys (*N* = 2) (Figs. 3 and S11). The injection site spanned the medio-lateral extent of Crus IIp, extending into the fissure between Crus IIp and Crus IIa. We set the survival time to allow the virus to infect first-order neurons, including neurons in the pontine nuclei^43^ and the inferior olive (Fig. S12), and then second-order neurons that project to the first-order neurons, including neurons in Layer V of the cerebral cortex (Figs. 3 and S13) and in the subthalamic nucleus^43^. The first-order inputs from the inferior olive to our injection sites originated almost exclusively from the lateral and ventral lamella of the principal olive (PO) (Fig. S12). These data indicate that our injections primarily involved cerebellar longitudinal zone D2^44^ that receives inputs from the ventral lamella of the PO^45^. To quantify the second-order inputs from the cerebral cortex, we examined coronal sections, spaced every 200 µm, and charted cortical neurons infected with rabies virus (*n* = 4889 in AB1, 10088 in AB2). No infected neurons were located outside of Layer V, confirming that the virus transport was restricted to the second-order neurons that are the origin of the cerebro-ponto-cerebellar pathway. A majority (63%) of the infected neurons in the two animals were located in regions of the prefrontal cortex (Fig. 3b). Additional populations of labeled neurons were found in the posterior parietal, posterior cingulate and retrosplenial areas (24%), and in regions of the insula and temporal cortex (10%). In contrast, limited numbers of infected neurons were present on the orbital surface (1%) and in cortical areas involved in motor control, including the primary motor cortex, premotor areas, and the frontal eye field (2%).

Approximately half of the neurons labeled in the prefrontal cortex were found in a single cortical area, termed the pre-dorsal premotor area (PrePMd), also referred to as F7^38^. Although immediately rostral to the dorsal premotor cortex (PMd), the PrePMd is not considered to be one of the cortical motor areas because it does not project directly either to the primary motor cortex (M1)^46^, or to the spinal cord. The PrePMd is interconnected with the PMd, but it receives its strongest cortical inputs from other areas of the prefrontal cortex^47,48^. Other prefrontal areas that contained infected neurons included areas 8 (11%), 9 l (3%), 10 l (3%), 46 (3%), and 12/45 (6%), as well as prefrontal areas on the medial wall of the hemisphere (8%) To determine density of labeling, we counted infected neurons in successive 200-µm × 200-µm bins. The densest bins in this sample (upper 90th percentile) contained 5 or more infected neurons (dark blue squares in Fig. 3b). This analysis indicated that 96% of the densest bins were located in the PrePMd. Thus, the highest density of inputs to the posterior-lateral cerebellum originated from the PrePMd.

**Fig. 4:**
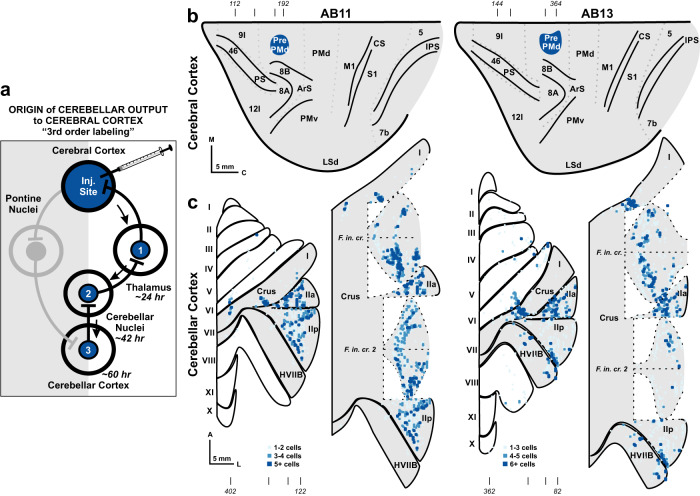
Origin of cerebellar cortex output to the PrePMd. **a** The schematic illustrates the experimental design and rabies virus transport in the cerebro-cerebellar circuits from injections into the PrePMd. Regions containing rabies virus infected neurons are shown in blue. Virus is transported retrogradely from the injection site in the cerebral cortex to first-order neurons, such as neurons in the thalamus. We estimate that this first stage of rabies transport takes approximately 24 h. The virus is then transported transsynaptically in the retrograde direction to second-order neurons and labels neurons in the deep cerebellar nuclei. At a survival time of 60 h, we observed neurons labeled by a further step of retrograde transneuronal transport to third-order P cells in the cerebellar cortex. **b** Dark blue outlines indicate the extent of the cerebral cortex injection sites in two cases (AB13 – Right; AB11 – Left) on flattened maps of the lateral wall. Estimate cytoarchitectonic boundaries are shown in light gray dotted lines. Tick marks (top) indicate level of sections depicted in Fig. S14. **c** The distribution of virus infected P cells following rabies virus injections into the PrePMd is illustrated on flattened surface maps of the cerebellar cortex in two cases (AB13 – Right; AB11 – Left). Colored squares on the flattened cerebellar cortical indicate the number of infected P cells in successive 200-µm × 100-µm bins (color key). Tick marks (bottom) indicate level of sections depicted in Fig. S15. Abbreviations as in Fig. 3.

Next, we injected the N2c strain of rabies virus into the center of the PrePMd in another set of cebus monkeys (*N* = 2) (Figs. 4 and S14). We adjusted the survival time to allow the virus to infect first-order neurons that project to the injection sites, including neurons in the thalamus, second-order neurons in the deep cerebellar nuclei, and third-order neurons (P cells) in the cerebellar cortex (Figs. 4 and S15). We examined sagittal sections through the cerebellum, spaced every 200 µm, and charted the infected P cells (*n* = 6962 in AB11, 6812 in AB13). The overwhelming majority (91.7%) of the infected P cells in both monkeys were located in portions of Crus I and II. Some common sites in Crus I and II of both animals contained dense patches of labeled P cells. These patches of labeled neurons were interspersed with areas that contained no labeled neurons. For example, the surface of Crus I, the posterior bank of the fissure between Crus I and IIa, and the surface of Crus IIa contained dense patches of labeled P cells in both animals.

In prior experiments we examined the distribution of P cells labeled following retrograde transneuronal transport of rabies virus from injections into prefrontal area 46 and M1^19^. We were able to compare our current results with those from the prior experiments. This comparison indicated that P cells projecting to area 46 are located at cerebellar sites that are largely separate from those projecting to the PrePMd. Even at a site of potential overlap on the surface of Crus IIa, the densest patches of P cells that project to area 46 are located largely lateral to those that project to the PrePMd. Similarly, P cells that project to M1 are located in more anterior regions of the cerebellar cortex that are clearly separate from those that project to the PrePMd. Thus, our neuroanatomical tracing results indicate that the cerebellar site where inactivation interferes with learning new visuomotor associations is not interconnected with any of the cortical motor areas, but forms closed-loop circuits with the prefrontal cortex.

## Discussion

We previously showed that the SS of Purkinje cells in Crus I and Crus II produce an error signal driven by the presence or absence of reward on the prior trial when monkeys learn a new visuomotor association. This reward-driven error signal approaches zero as the monkeys learn the task. When the monkeys perform an overtrained visuomotor association task which does not require learning, the same neurons are not responsive to reward or error. Here, we show that reversible inactivation of specific regions of the primate posterior-lateral cerebellar cortex (Crus I and II), which form closed-loop circuits with the prefrontal cortex, interferes with learning new visuomotor associations. Inactivation of this non-motor area of the cerebellum affects neither performance of the task with well-practiced associations, nor movement parameters. Thus, our results indicate that non-motor regions of the primate cerebellar hemisphere may use reward signals^39,49^ to shape the neural activity in interconnected regions of the prefrontal cortex, promoting conditional associative learning.

The ability to learn and perform arbitrary visuomotor associations flexibly engages a brain-wide network, including areas of the premotor and prefrontal cortex, as well as interconnected regions of the basal ganglia and the hippocampus^50^. Multiple studies provide support for anatomical and functional distinctions between the dorsal premotor cortex (PMd) and the PrePMd, a cortical area immediately rostral to the PMd. The PrePMd does not project directly to the primary motor cortex (M1)^46^ or to the spinal cord^51^. Thus, it can be argued that the PrePMd is anatomically more similar to areas of the prefrontal cortex^38^, with which it is closely interconnected^47,48^. Neurophysiology studies in nonhuman primates and neuroimaging studies in humans also support a functional distinction between the PrePMd and the PMd: activity in the PrePMd is more closely related to higher-order cognitive functions, including learning of conditional visuomotor associations^38,52–55^. Indeed, lesions that primarily involve the PrePMd cause severe deficits in the acquisition of visuomotor associations^56^.

Patients with cerebellar pathology also show impairments in associative learning, including learning of arbitrary visuomotor associations^57,58^. However, the cerebellum has not been considered essential for the function of the brain-wide network for learning visuomotor associations^50,59–61^. To the contrary, previous causal experimental studies have not found convincing evidence for cerebellar contributions to learning arbitrary visuomotor associations^50,62–64^. The prior attempts to identify a causal role for the cerebellum in learning visuomotor associations have used broad and irreversible lesions that included the deep cerebellar nuclei and overlaying cerebellar cortex^62–64^. This broad approach involves permanent lesions of both motor and non-motor regions of the cerebellum^23^, thus complicating the behavioral effects and the interpretation of results. In contrast, in the present experiments we targeted reversible inactivation to the specific regions of the cerebellar cortex (Crus I and II) that are interconnected with the PrePMd and where we previously recorded reinforcement error signals during learning of new visuomotor associations^39^.

Recent studies in rodents highlight the involvement of Crus I and II in various functions including preparatory motor activity, sensorimotor integration, accumulation of sensorimotor evidence, eye-blink conditioning, social behavior, and decision making based on sensory discrimination^64–71^. In rodents, Crus I and II are interconnected with both sensorimotor and non-motor regions of the cerebral cortex^24,66,72^. In nonhuman primates^19,20^ and humans^28,73–76^, Crus I and II appear to be predominantly interconnected with regions of the prefrontal cortex. There is considerable evidence for evolutionary expansion of cerebellar regions that are interconnected with the prefrontal cortex in primates^2^ and the brain-wide circuits in which these regions participate may have limited homologs in rodents^77^. Further studies are needed to help reveal the equivalent brain-wide circuits for associative learning in rodents.

To understand the role of the cerebellum in the brain-wide circuit that uses reward information to guide associative learning, we must consider the sources of its reward-related inputs. We have previously shown that the same region of cerebellar cortex that is involved in learning visuomotor associations^39^ and is interconnected with the PrePMd (Figs. 3 and 4), also receives di-synaptic inputs from associative regions of the subthalamic nucleus (STN) in the basal ganglia^43^ (Fig. S16). Neurons in both the prefrontal cortex and the basal ganglia are selective for reward or failure on the prior trial^78^ although these signals do not change during learning. Thus, signals from both the prefrontal cortex, including the PrePMd (Fig. 3), and the basal ganglia^43^, could convey reward-related signals to the posterior-lateral cerebellum through mossy fiber inputs to the pontine nuclei^79^, or through the meso-diencephalic junction (MDJ) inputs to the inferior olive^45,80^, the two primary input routes to the P cells of the cerebellar cortex (Fig. S16). Granule cells have been recently shown to carry reward information while performing an overlearned task^81^, but it is unknown how they process reward during reinforcement learning. During reinforcement learning, complex spikes do not sculpt concurrent simple spike responses^37^, as posited in theories of cerebellar contributions to cognition based on the classical Marr-Albus error-based motor learning framework^11,12^, nor do they provide trial-by-trial information about reward, although they do signal the probability of success on the current trial. Instead, complex spikes may provide a motivational signal^37^ through cerebellar projections to the dopaminergic midbrain that have been demonstrated in mice. In mice, complex spike activity could signal reward prediction errors^82,83^ and temporal difference prediction errors^84,85^. The reinforcement learning error signal encoded in the posterior-lateral cerebellar P cell simple spikes is present when the monkeys start to learn a new visuomotor association and decreases as they learn the task. This signal could provide the bulk of the neural substrate for reinforcement learning at the level of the cerebellar cortex. The climbing fiber and about 50 P cells synapse onto a single deep cerebellar (DCN) neuron^86^. Both motor and non-motor regions of the cerebellum send di-synaptic outputs to the basal ganglia^87^. Therefore, the phasic reinforcement error signal carried by the P cells could be relayed by downstream DCN neurons (Fig. S16) to regions of the prefrontal cortex, including the prePMd, as well as to regions of the basal ganglia, and provide tonic informative signals regarding the outcome of prior decisions. Although neurons in both the prefrontal cortex and the associative striatum are selective for reward or failure on the prior trial^78^, these cells do not change their activity with learning, unlike P cells in the lateral-posterior cerebellum. In the lateral-posterior cerebellum, the difference in activity between reward- and error-selective P cells approach zero as the monkey learns the task. Thus, the synergy between the contributions from the cerebellum to prefrontal circuits with the basal ganglia may provide the flexibility necessary for learning complex cognitive functions and optimizing cognitive rules.

One powerful way to test this is through reversibly inactivating specific nodes of the brain-wide circuit to study that node’s contribution to reinforcement learning. Lesions in prefrontal cortex of the monkeys eliminates their ability to learn a new visuomotor association and impairs their ability to retain associations learned preoperatively^88^. Furthermore, dopamine receptors in the prefrontal cortex are necessary for learning new visuomotor associations^89^. Combined lesions of basal ganglia and prefrontal cortex prevent monkeys from retaining associations learned preoperatively, but does not affect their ability to learn new associations^62^. Here, we provide conclusive evidence for a causal cerebellar contribution to learning arbitrary visuomotor associations, by reversibly inactivating the specific region of cerebellar cortex where we recorded reinforcement learning error signals^39^. These results provide support for the notion that interfering with any node in the cerebro-cerebellar-basal ganglia network has important consequences for its function^90^ and help solidify a causal role for the non-motor cerebellum in reinforcement learning.

Contemporary research has led to the discovery of new ways in which cerebellum may enable different types of learning and suggest that we need to reconsider whether the cerebellum implements uniform computations across different functional domains^91^. The remarkably uniform organization of cerebellar microcircuitry has inspired the “universal cerebellar transform” hypothesis^92^. Although the cerebellum appears to have a generally uniform microcircuitry, heterogeneity can be found at various levels^93^, including protein expression^94–96^ and local microcircuitry^97,98^. At the level of P cells, we have shown differences in functional properties: some P cells that report previous correct trials and others report previous wrong trials^39^. During motor learning, others have shown that P cells can be bursters or pausers^99^. At the level of learning mechanism, the complex spikes encode motor errors and can teach the simple spikes to correct the behavior^100,101^, but that is not always the case^102^. Simple spikes can also directly encode motor^103,104^ and non-motor^39^ errors, or may receive teaching signals from other components of the cerebellar microcircuits^105^. Our results suggest that motor and non-motor regions of the cerebellum may differentially process reward information. In the eye-movement related cerebellar floccular complex, information about reward is already transformed into motor commands^36^. Here, we have shown that reward information in the non-motor cerebellum plays an active role in shaping the learning of arbitrary visuomotor associations. These findings provide evidence supporting “multiple functionality” in the cerebellum^91^ and suggest that cerebellar circuits may implement functionally distinct computations to enable flexible cognitive learning and behavior.

## Methods

### Experimental modeland subject details

We performed all behavioral and inactivation experiments on two adult male monkeys (*Macaca mulatta*), B and S, weighing 10–11 kg each. All physiological, behavioral and inactivation experimental protocols were approved by the Animal Care and Use Committees at Columbia University and the New York State Psychiatric Institute and complied with the guidelines established by the Public Health Service Guide for the Care and Use of Laboratory Animals. All monkeys were lab bred.

The anatomical results are based on observations from four adult cebus monkeys (*Cebus apella*, 3 female, 1 male, 1.74–3.4 kg, 3–16 years old). We used cebus monkeys because, unlike Old World primates, this species is susceptible to infection with the herpes simplex virus type 1 (HSV1), an anterograde transsynaptic tracer that we have previously used to reveal the closed-loop architecture of cerebro-cerebellar circuits^19^. In our experience, we have observed no differences in the macroarchitecture of cerebro-cerebellar circuits between cebus and macaque monkeys.

In each monkey, we injected a mixture of the N2c strain of rabies virus (CVS-N2c 4.5 × 10^9^ pfu/mL) and 0.02% of a conventional tracer (beta-subunit of cholera toxin, CTb) into the cerebellar (*N* = 2) or cerebral (*N* = 2) cortex. All anatomical tracing procedures were conducted in accordance with the Association for Assessment and Accreditation of Laboratory Animal Care and the National Institutes of Health Guide for the Care and Use of Laboratory Animals. The experimental protocol was approved by the Institutional Animal Care and Use Committee and the Biosafety Committee at the University of Pittsburgh. Biosafety practices conformed to the Biosafety Level 2 regulations outlined in *Biosafety in Microbiological and Biomedical Laboratories* (Department of Health and Human Services publication no. 93-8595). Details of our procedures for handling rabies virus and virus infected animals have been previously published^106^.

### Method details

#### Behavioral task

We used the NIH REX-VEX system for behavioral control^107^ and stimulus presentation. The monkey sat inside a dimly lit recording booth, with its head firmly fixed, in front of a Samsung video monitor.

The task began with the monkeys grasping two bars, one with each hand, after which a white 1° × 1° square appeared as a trial cue for 800 ms. Then one of a pair of symbols (6° × 6° square) appeared at the center of gaze for 100 ms. One symbol signaled the monkey to release the left bar and the other to release the right bar. We rewarded the monkeys with a drop of juice for releasing the hand associated with that symbol. The liquid reward and a beep paired with opening of the solenoid were delivered immediately (with a delay of 1 ms) after the initiation of the correct hand movement. The kinematics or the dynamics of the actual hand movement made by the monkeys thereafter were irrelevant and did not affect the reward delivery (although the subjects made well-learned stereotypic hand movements).

We trained the monkeys to associate a specific pair of symbols (green square and pink square) with specific actions (left and right-hand release, respectively) for about 4–6 months until their performance was above 95% correct; we refer to this as the overtrained task.

In the visuomotor associative learning version of the task, we began every recording session by presenting the monkeys with the same overtrained symbol pair (overtrained task), and after a number of trials, switched the symbol pair to two fractal symbols, which the monkey had never seen before (novel condition, see Fig. S1a for the library; we used 16 pairs for monkey B and 9 pairs for monkey S from the same library).

Our main aim was to test the effect of muscimol on learning new visuomotor associations and use saline infusions as a control. Therefore, using different symbols for saline vs muscimol infusions could add yet another confounding variable: difficulty of the association. It is well known that monkeys take a relatively longer time (more trials) to learn more difficult association. Therefore, we wanted to study the effect of saline and muscimol treatments on the same symbol pairs. This required us to come up with a new task design.

First, we wanted to get a robust estimate of the difficulty of each symbol pair for each monkey. Since different symbol pairs in the library had different levels of complexities and similarities, the time (number of trials) each monkey took to learn the association differed among the different symbol pairs. Therefore, we recorded each monkeys’ performance for each symbol pair (Monkey B- 16 pairs, Monkey S- 9 pairs) at least three times, spread over several weeks, presented in random order (Fig. 1c). To prevent the monkeys from relying on their previous experience with the same symbol pair to re-learn their associations when we presented the same symbol pairs again, we either presented a given symbol pair again only several days after its prior presentation, with intervening presentations of other symbol pairs or random pairs not from the library (Fig. 1c; symbol pair highlighted in blue), or we reversed the symbol-hand association the next time we presented the same pair (Fig. 1c; symbol pair highlighted in orange).

The acquisition-difficulty levels (the trial when they reached 90% correct for each repetition of each symbol pair) were not significantly different among the repeated presentations of the symbol pair (Fig. S1b; monkey B: *P* = 0.46; monkey S: *P* = 0.63, ANOVA), suggesting that the monkeys forgot their prior experience with a given symbol pair during each additional re-learning.

Furthermore, the area between two randomly chosen learning curves for the same symbol was significantly lower than the area between two randomly chosen learning curves across two different symbols (normalized by the length of the shortest learning curve; Fig. S1f, g). That is, when the same symbol was presented again, the learning behavior was similar, even though they relearned the association without interference from the memory of previous learning.

We used two versions of the visuomotor associative learning task. In the more difficult version (*N* = 16 for monkey B and *N* = 9 for monkey S), the symbols appeared with equal probability regardless of the monkey’s prior decision (Fig. 1). In the easier version, we used an error-correction strategy, showing the same symbol repeatedly in the next trial until the monkey made a correct decision (Fig. S4a–c; *N* = 3 for monkey B and *N* = 5 for monkey S). Monkeys learned the symbol-hand association faster during this paradigm, under control conditions (Fig. S4–g). However, this task did not have any effect on the performance of overtrained task (Fig. S4k). The manipulanda remained the same throughout the task. We did not use data from error-correction experiments in any of our other analyses. In total, both the monkeys performed ~7500 trials on an average (25 pairs × 3 repetitions per pair × ~100 trials per learning).

In the manipulanda change task (Fig. S8; *N* = 3 for monkey B and *N* = 3 for monkey S), we began every recording session by presenting the monkeys with the same overtrained symbol pair and bar manipulanda, and after a number of trials, switched the bar manipulanda to dowel manipulanda. The visuomotor association remained the same.

In all these paradigms, a correct trial was defined as the trial in which the monkey released the correct hand associated with the symbol. Note that since we initiated the reward 1 ms after the monkeys broke contact with the correct bar with the responding hand and continued to not have any contact for 50 ms while they maintained contact with the incorrect bar (with the non-responding hand) for the same interval, the kinematics or the dynamics of the actual hand movement made by the monkeys thereafter were irrelevant and did not affect the reward delivery. The monkeys received reward only for correct trials. We defined a wrong trial as the trial in which the monkey released the hand not associated with the symbol. Trials where the monkeys released both hands anytime during the trial or released the hand(s) before the symbol onset or released the hand(s) after 2800 ms from symbol onset were considered abort trials and were neither rewarded nor analyzed. The aborted trials contributed to <7% of all saline trials and <9% of all muscimol trials across all sessions.

#### Analysis of the learning behavior

We constructed the learning curve for every session by calculating the percent correct trials in a sliding window of 10 trials as the bin width moved by 5 trials. Then, we averaged the learning curves across repeated sessions for each symbol pair and separately for each monkey (16 for monkey B and 9 for monkey S) to obtain a total of 25 learning curves. We estimated when the performance first reached above 90% and referred to it as the acquisition-difficulty level for each session. We repeated this for both saline-control condition and the muscimol-inactivation condition. If the monkeys reached >90% correct through the above method and remained above 80% for at least the next 20 trials, the associations were considered ‘learned’. Monkeys usually performed about 100–125 trials in total. They often tended to lose motivation or succumbed to fatigue after this and started to make errors (Fig. S2c). Therefore, in this study, we only report the first 100 trials of learning novel symbols.

#### Drift-diffusion reinforcement learning model

We developed a computational model combining a drift-diffusion framework for choice discrimination and reinforcement learning to predict the monkey’s behavioral performance given the cerebellar neural activity^39^. Briefly, we model the action selection (of action choices, ${a}_{L}$ and ${a}_{R}$ corresponding to left and right-hand bar-release hand movements respectively) through a race to threshold model where there is a race in the evidence accumulation between the action values ${a}_{L}$ and ${a}_{R}$ modeled by Wiener first-passage time (WFPT) distribution:

1 $${V}_{i}\left(x\right) \sim {{{{{\rm{WFPT}}}}}}\,\left[{\upsilon }_{i}\left(t\right)\right]$$

where $\upsilon \left(t\right)$ denotes the rate of accumulation process for the trial $t.$The rate of accumulation in the overtrained (OT) condition, ${\upsilon }_{i}^{{{{{{\rm{OT}}}}}}}$ is assumed to be a constant. Then, we model the evolution of ${\upsilon }_{i}\left(t\right)$, with learning as:

2 $${\upsilon }_{i}\left(t+1\right)={\upsilon }_{i}\left(t\right)+\left(\frac{m}{r}\right){\Delta }_{i}^{{{{{{\rm{CB}}}}}}}\left(t\right)\,{I}_{c}\left(t\right)\,{I}_{i}(t)$$

where:

3 $${\Delta }_{i}^{{{{{{\rm{CB}}}}}}}\left(t\right)=r\,\left[{\upsilon }_{i}^{{{{{{\rm{OT}}}}}}}-{\upsilon }_{i}\left(t\right)\,\right]\,{I}_{c}\left(t\right)\,{I}_{i}\left(t\right)+({I}_{w}\left(t\right)+{I}_{c}\left(t\right)\,{I}_{i\ne j}\left(t\right))\,{\Delta }_{i}^{{{{{{\rm{CB}}}}}}}\left(t-1\right)$$

here, ${m}$ is the rate of learning, $r$ is the scale factor, ${I}_{i}\left(t\right)$ is the indicator function describing the chosen action (${a}_{{{{{{\rm{ch}}}}}}}$) on trial ${t}$ defined as follows:

4 $${I}_{i}(t)=\left\{\begin{array}{c}1\,{{{{{\rm{if}}}}}}\,{a}_{{{{{{\rm{ch}}}}}}}(t)={a}_{i}\\ 0\,{{{{{\rm{if}}}}}}\,{a}_{{{{{{\rm{ch}}}}}}}(t) \, \ne \, {a}_{i}\end{array}\right.$$

We previously showed the rate of change of the accumulation rate ${\upsilon }_{i}\left(t\right)$, given by ${\Delta }_{i}^{{{{{{\rm{CB}}}}}}}\left(t\right)$ is represented in the delta epoch, the period of time for each neuron in which the reinforcement learning error signal occurs, of the P-cell population that fired at the time of interest^39^. We estimated the accumulator’s initial and final rates by fitting a drift-diffusion model to the observed RT values. Then, we minimized the error between the generated and the empirical RT distributions for the OT and initial learning tasks to get estimates of the parameters. We then simulated the learning process with these values. For cerebellar inactivation condition, we modeled the firing rate of P cells in the delta epoch, ${\Delta }_{i}^{{{{{{\rm{CB}}}}}}}\left(t\right)$ to be a random Gaussian noise and repeated all the above steps otherwise identically (Fig. S3b, c).

#### Magnetic resonance imaging and identification of cerebellar areas

We first performed MRI in 3 T Siemens MRI scanner using a Kopf MRI compatible stereotaxic apparatus with fiducial markers secured to the head post of the monkeys. Then, using Brainsight (Rogue Research), we mapped the MR images to the fiducials offline and then localized the different cerebellar lobules in both monkeys. We adapted procedures for unfolding the cerebellar cortex from histological sections^19^ to construct unfolded maps of muscimol injection sites, using MRI images, custom laboratory software, and cerebellar nomenclature according to Larsell^108^ (Figs. 1d, S9a, and S10).

#### Single unit recording

Briefly, we used two recording cylinders, on the left hemisphere of each monkey. To insure that we were injecting muscimol into an appropriate region, and that the muscimol suppressed the neural activity, we introduced an injectrode which had a glass-coated tungsten electrode with an impedance of 0.8–1.2 MOhms (FHC) and a Hamilton syringe for infusion (see below), into the lateral-posterior cerebellum of monkeys (monkey B: only the left side; monkey S: some sessions in the left and some sessions in the right sides but not simultaneously) every day that we recorded using a Hitachi microdrive. We passed the raw voltage signal recorded from the electrode through a FHC Neurocraft head stage, and amplifier, and filtered through a Krohn-Hite filter (bandpass: lowpass 300 Hz to highpass 10 kHz Butterworth), then through a Micro 1401 system, CED electronics. We used the NEI REX-VEX system coupled with Spike2 (CED electronics) for event and neural data acquisition. We verified all recordings offline to ensure that we had isolated P cells and that the spike waveforms had not changed throughout the course of each experimental session^37^.

#### Muscimol infusion protocol

At the beginning of each day, we lowered a cannula filled with 10 μL of either saline or 10 μg/μL muscimol (diluted in 1× phosphate buffered saline solution), in a track selected on the basis of single unit electrophysiological recordings of task related P cells near Crus I and II of the lateral-posterior cerebellum. We slowly infused the solution using a syringe pump (Harvard Apparatus) through a direct line to the cannula at a constant rate (0.2–0.5 μL/min for 20–30 min). We delivered a total mass of 4–10 μg of muscimol in each session. Previous studies have reported that muscimol diffuses and hence functionally inactivates the neurons in an estimated span of 2–3 mm of spherical radius^109,110^. Muscimol infusions (10 for Monkey B and 4 for Monkey S in lateral-posterior cerebellum and 1 for Monkey B and 3 for Monkey S in the lateral anterior cerebellum) were typically made at different depths within a single track (total span of ~5 mm) to increase the diffusion radius of the chemical. The same tracks were used to saline injections. After infusion, the cannula was left for at least 20–30 min in situ. We started behavior recordings for the day only after 30–40 min after the injection (Fig. 1e). We only injected either saline or muscimol on a given day and never both on the same day. Furthermore, after each muscimol injection, we waited at least 24–36 h before another injection (of saline or muscimol) so that muscimol is completely cleared off from the body.

#### Verification of the infusion sites

We mapped the injections sites on to a flattened map and confirmed our target locations (Figs. 1d and S9a). Lateral-posterior cerebellar inactivation often presented with slower reaction times and lateral anterior cerebellar inactivation presented with nystagmus in addition to modestly slower reaction times (Fig. S9g, h). Saline injections did not silence the neurons (Fig. 1e), nor produced perceivable changes in gross behavior.

One potential concern with our injections was that our treatment could have affected the visual cortex right above the cerebellum leading to visual deficits, resulting in monkeys’ poor performance. However, this could not be the case. First, the multiple penetrations into the cerebellum resulted in hemorrhages through the overlying visual cortex (as seen from the MRIs in Figs. 1d and S9a). This part of the cortex represents the inferior 2–20 degrees of the contralateral visual field. Although the hemorrhages occurred well before we began the anterior injections, inactivation of anterior cerebellum, which occasionally caused nystagmus and leg ataxia did not affect the monkeys’ ability to learn new visuomotor associations. The ipsilateral visual field was intact, and the monkeys were not forced to fixate, so the deficit (even if it had occurred) would not have been a problem for the monkeys to see the symbols. Although diffusion above the needle track is always possible, it was highly unlikely that the muscimol would have diffused into the brain above the tentorium cerebelli, because the guide-tube track went through the cistern overlying the cerebellum, and would have diffused into cisterns, which has less barrier to diffusion than brain tissue. Finally, if muscimol had diffused into the hemorrhage sites it was highly unlikely that it would have further diffused into active visual cortex.

#### Effect of different treatments on behavior

Since different symbol pairs had different acquisition-difficulty levels (Fig. S1b–d), we compared the learning behavior for the same pair of symbols in both saline and muscimol conditions. To do this, for each presentation of a symbol pair, we computed the learning curves for saline and muscimol injections. and identified three trials which bracketed 50%, 60%, 70 %, 80 %, 90 % and 100% correct level in the saline condition, and then measured the percent correct in the muscimol-inactivation sessions for the same symbol pairs on these same trials identified from the saline sessions. Clearly, for each symbol pair, we identified the first 3 trials whose average the probability of learning was $p$ where $p$ ~ 0.5, 0.6, 0.7, 0.8, 0.9 and 1.0. We denote this as $\Pr ({L}_{s}=p)$, where ${L}_{s}$ is the random variable associated with learning under saline condition. We then measured the probability of learning in the inactivation sessions for the same symbol pairs on these same set of trials identified from the control sessions. That is, we calculated the conditional probability of learning in the inactivation sessions, given the probability of learning in the control sessions ($\Pr ({{L}_{m}{|\; L}}_{s}=p)$. Therefore, for each given quantile of performance during control condition, this measure tells us the performance during inactivation condition on the same trials for the same pairs (Fig. 1h, i).

For lateral-posterior cerebellar inactivation, as the performance of the monkeys improved during control sessions, the performance of the monkeys also improved during inactivation sessions, but with a much slower rate (Fig. 1h, i). That is, ($\Pr \left({{L}_{m}{|\; L}}_{s}=p\right) \ll \, {Pr}\left({L}_{s}=p\right)\forall p\in [0.5,\, 1.0]$. However, for the lateral anterior cerebellar inactivation, the conditional probability of learning in the inactivation session given the probability of learning in the saline condition was comparable to the probability of learning in the control session (Fig. S9d, e). That is, ($\Pr \left({{L}_{m}{|\; L}}_{s}=p\right)={Pr}\left({L}_{s}=p\right)\forall p\in [0.5,\, 1.0]$.

The learning performance during cerebellar inactivation was independent of the rate of learning of a symbol pair in the control session for both monkeys. That is, when the lateral-posterior cerebellum was inactivated, the monkeys had difficulty learning even the association that had the least acquisition difficulty (Fig. S1e). In stating this result, it is critical to rule out the possibility that the image complexity of the symbols affected learning during inactivation. The symbol library (Fig. S1a) contained symbols with varying image complexities. The monkeys did not perform more than 80% even on symbol pairs with very low fractal image complexities (for example, one or two hues dominating the entire image).

Finally, we did not observe any systematic relationship between the concentration of the infused muscimol or saline and the deficit in learning behavior. However, relatively higher concentrations of muscimol impaired the learning slightly more (Fig. S2d).

#### Hand tracking

We painted a spot on the monkeys’ right hand with a UV-blacklight reactive paint (Neon Glow Blacklight Body Paint) prior to every session (Fig. S8a, c). We used a 5 W DC converted UV blacklight illuminator to shine light on the spot. Then we used a high speed (250 fps) camera (Edmund Optics), mechanically fixed to the primate chair, to capture a video sequence of the hand movement while the monkeys performed the tasks. We used the track mate Image J^111,112^ and custom written software in MATLAB to semi-manually track the fluorescent paint spot painted on the monkey’s hand. Since the P cells in the lateral-posterior cerebellum had comparable activities for ipsi and contra hand movements^39,113^ we only recorded one hand’s movement. Any effect a manipulation might have on hand movement behavior must be seen in both hand movements similarly.

#### Licking

We recorded licking at a sampling rate of 1000 Hz using a capacitive touch sensor coupled to the metal water spout which delivered liquid water reward near the monkey’s mouth. Raw binary lick traces were used to generate instantaneous lick rate by trial averaging and smoothing it with a Gaussian kernel of sigma = 20.

#### Eye movements

We tracked the monkey’s left eye positions at 240 Hz sampling rate with an infrared pupil tracker (ISCAN, Woburn, MA USA) interfaced with Spike 2 (CED electronics) where it was upsampled to 1000 Hz and synced with the event markers from NEI REX-VEX system^114,115^. The monkeys were free to move their eyes and were not restricted in any way. They made various non-task related eye movements were not controlled for, although they tended to look at the symbols when they appeared. Therefore, the eye movements for consecutive trials for the same symbols were highly variable^116,117^ (Fig. S6e). Therefore, we indexed the eye movements, through radius of visual exploration (*r*_exp_). To calculate this, we first created a 2D spatial density histogram from all the overlaid eye movements (see Fig. S6f for a single trial example and Fig. S6g, h for population). We then fit a 2D Gaussian distribution to this data to measure the extent of spread from the center and calculated the full-width-at-half-maximum. We did not see any consistent pattern of microsaccades, although the 0.5° accuracy of our eye tracking system made it difficult to monitor smaller microsaccades.

#### Statistics

To check if two independent distributions were significantly different from each other, we first performed a two-sided goodness of fit Lilliefors test, to test for the normality, then used an appropriate t-test; or else a non-parametric Wilcoxon Mann–Whitney *U* test. All paired tests were two-tailed. All values in this study (including the shadings and the error bars in the figures), unless stated otherwise, are mean ± s.e.m.

#### Rabies virus tracing

All surgical procedures for rabies virus injections were performed under aseptic conditions. The night before virus injection surgery, monkeys were administered dexamethasone (0.5 mg/kg IM). The morning of surgery the monkeys were fasted for 2–6 h and sedated with ketamine (15 mg/kg IM), intubated and maintained on gas anesthesia (isoflurane 1–3% vol/vol). Dexamethasone (0.5 mg/kg IM), glycopyrrolate (0.01 mg/kg IM) and an antibiotic (ceftriaxone 75 mg/kg IM) were administered at the time of surgery. Respiratory rate, blood oxygen level, body temperature, and sensitivity to noxious stimuli were monitored at regular intervals during the procedure. Each monkey had its head restrained in a Kopf stereotaxic frame (Kopf Instruments). We performed a craniotomy to expose either (1) the ventral portion of the occipital cortex and the lateral aspects of the posterior cerebellum or (2) the lateral prefrontal cortex. We resected the dura and used a Hamiton syringe (30-gauge needle) to place multiple injection tracks into either the cerebellar hemisphere (Crus IIp) or the PrePMd. We injected small amounts (0.2 µl) of a mixture of rabies virus (CVS-N2c 4.5 × 10^9^ pfu/mL; provided by M. Schnell) and 0.02% CTb (List Biological Laboratories) at depths at least 0.5 mm apart along the injection tracks. Craniotomies and depths of injection tracks were based on structural magnetic resonance images previously acquired in each monkey. After completion of virus injections, the craniotomies were covered with artificial dura and the incisions were closed in anatomical layers. The monkeys were place in an isolation chamber and administered an analgesic (buprenorphine 0.01 mg/kg IM) and desamethasone (0.25 mg/kg IM) every 8–12 h for the duration of the survival time, or 48 h. Based on our experience with the N2c strain of rabies virus, we set the survival time to 42 h following cerebellar cortex injections to allow for second-order transport (Fig. 3) and to 60 h following the cerebral cortex injections to allow for third-order transport (Fig. 4).

At the end of the survival time, monkeys were deeply anesthetized using ketamine (25 mg/kg IM) and an overdose of pentobarbital sodium (40 mg/kg IV or IP). Monkeys were perfused transcardially with 0.1 M phosphate buffer (pH 7.4), followed by a series of fixatives (10% buffered formalin, followed by 10% buffered formalin with 10% glycerol at 4 °C). The brain was extracted and stored overnight in 10% buffered formalin with 10% glycerol at 4°C, and then switched to 10% buffered formalin with 20% glycerol at 4 °C for 1–2 weeks. Blocks of tissue (cerebral cortex, brainstem, and cerebellum) were individually frozen and sectioned at 50 µm on a cryostat. The cerebral cortex was blocked anterior to the parietal-occipital sulcus and cut in the coronal plane. The cerebellar cortex was blocked through the paravermis and cut in the sagittal plane. Every 10th section was stained with cresyl violet for cytoarchitecture analysis. Brain sections were immunohistochemically stained according to the avidi-biotin peroxidase method (Vectastain; Vector Laboratories). We used a mouse monoclonal antibody (clone M957) that targets the rabies virus phosphoprotein^118^ (Rabies Centre of Expertise, Canadian Food Inspection Agency, Canada; dilution 1:300) to detect rabies virus. We use the goat anti-Cholera Toxin B Subunit (List Biological Laboratories, Campbell CA; Cat #703, RRID:AB_10013220; dilution 1:10,000) antibody to detect CTb. Stained tissue sections were mounted on glass slides, air dried, and coverslipped with Cytoseal.

Every fourth section through the cerebral cortex and the cerebellum were examined for immunostaining for rabies virus using bright field and polarized illumination. Every fourth sections through the cerebellar cortex and cerebral cortex were examined for CTb labeling to identify the extent of injection sites for our injections in the cerebellar and cerebral cortex, respectively. We plotted section outlines, injection site outlines or rabies labeled neurons, gray-white matter boundaries, cytoarchitectonic borders, and other anatomic features using a computer-based charting system (MD2 or MD3; Accustage). We created unfolded maps of the cerebral and cerebellar cortex that displayed the injection sites or the distribution of labeled neurons, as well as cortical sulci or cerebellar fissures on 2D surface maps of the cerebral and cerebellar cortex^19^. Reconstructions were performed using ReconWin 2020 (Great Island Software, Chatham MA). Cerebellar nomenclature is according to Larsell^108^.

### Reporting summary

Further information on research design is available in the Nature Portfolio Reporting Summary linked to this article.

### Supplementary information

Supplementary Information

Reporting Summary

## Data Availability

Supporting data and code for this study are available at https://github.com/naveen-7/Cerebellum-reward. A reporting summary for this article is available as a Supplementary Information file. Source data are provided with this paper.

## Code availability

The codes used for the analyses that support the findings of this study are available from https://github.com/naveen-7/Cerebellum_reward.

## References

[1] Smaers JB, Steele J, Zilles K (2011). Modeling the evolution of cortico-cerebellar systems in primates. Ann. N. Y. Acad. Sci..

[2] Balsters JH (2010). Evolution of the cerebellar cortex: the selective expansion of prefrontal-projecting cerebellar lobules. Neuroimage.

[3] Ramnani N (2006). The evolution of prefrontal inputs to the cortico-pontine system: diffusion imaging evidence from Macaque monkeys and humans. Cereb. Cortex.

[4] Kandel, E. R., Koester, J. D., Mack, S. H. & Siegelbaum, S. A. Principles of Neural Science, Sixth Edition (McGraw Hill, 2021).

[5] Asanuma C, Thach WT, Jones EG (1983). Distribution of cerebellar terminations and their relation to other afferent terminations in the ventral lateral thalamic region of the monkey. Brain Res..

[6] Hoover JE, Strick PL (1999). The organization of cerebellar and basal ganglia outputs to primary motor cortex as revealed by retrograde transneuronal transport of herpes simplex virus type 1. J. Neurosci..

[7] Eccles JC (1967). Circuits in the cerebellar control of movement. Proc. Natl Acad. Sci. USA.

[8] Thach WT, Goodkin HP, Keating JG (1992). The cerebellum and the adaptive coordination of movement. Annu. Rev. Neurosci..

[9] Ito M (2002). Historical review of the significance of the cerebellum and the role of Purkinje cells in motor learning. Ann. N. Y. Acad. Sci..

[10] Manto M (2012). Consensus paper: roles of the cerebellum in motor control–the diversity of ideas on cerebellar involvement in movement. Cerebellum.

[11] Marr D (1969). A theory of cerebellar cortex. J. Physiol..

[12] Raymond JL, Medina JF (2018). Computational principles of supervised learning in the cerebellum. Annu. Rev. Neurosci..

[13] Raymond JL, Lisberger SG, Mauk MD (1996). The cerebellum: a neuronal learning machine?. Science.

[14] Medina JF, Lisberger SG (2009). Encoding and decoding of learned smooth-pursuit eye movements in the floccular complex of the monkey cerebellum. J. Neurophysiol..

[15] Grodd W, Hulsmann E, Lotze M, Wildgruber D, Erb M (2001). Sensorimotor mapping of the human cerebellum: fMRI evidence of somatotopic organization. Hum. Brain Mapp..

[16] Bodranghien F (2016). Consensus paper: revisiting the symptoms and signs of cerebellar syndrome. Cerebellum.

[17] Flourens, P. *Expériences sur le système nerveux* (Chez Crevot, libraire-éditeur, 1825).

[18] Leiner HC, Leiner AL, Dow RS (1986). Does the cerebellum contribute to mental skills?. Behav. Neurosci..

[19] Kelly RM, Strick PL (2003). Cerebellar loops with motor cortex and prefrontal cortex of a nonhuman primate. J. Neurosci..

[20] Strick PL, Dum RP, Fiez JA (2009). Cerebellum and nonmotor function. Annu. Rev. Neurosci..

[21] Middleton FA, Strick PL (2000). Basal ganglia and cerebellar loops: motor and cognitive circuits. Brain Res. Brain Res. Rev..

[22] Hashimoto M (2010). Motor and non-motor projections from the cerebellum to rostrocaudally distinct sectors of the dorsal premotor cortex in macaques. Eur. J. Neurosci..

[23] Dum RP, Li C, Strick PL (2002). Motor and nonmotor domains in the monkey dentate. Ann. N. Y. Acad. Sci..

[24] Pisano TJ (2021). Homologous organization of cerebellar pathways to sensory, motor, and associative forebrain. Cell Rep..

[25] Middleton FA, Strick PL (2000). Basal ganglia output and cognition: evidence from anatomical, behavioral, and clinical studies. Brain Cogn..

[26] Argyropoulos GPD (2020). The cerebellar cognitive affective/schmahmann syndrome: a task force paper. Cerebellum.

[27] Buckner RL (2013). The cerebellum and cognitive function: 25 years of insight from anatomy and neuroimaging. Neuron.

[28] Buckner RL, Krienen FM, Castellanos A, Diaz JC, Yeo BT (2011). The organization of the human cerebellum estimated by intrinsic functional connectivity. J. Neurophysiol..

[29] Koziol LF, Budding DE, Chidekel D (2012). From movement to thought: executive function, embodied cognition, and the cerebellum. Cerebellum.

[30] Keren-Happuch E, Chen SH, Ho MH, Desmond JE (2014). A meta-analysis of cerebellar contributions to higher cognition from PET and fMRI studies. Hum. Brain Mapp..

[31] Stoodley CJ, Valera EM, Schmahmann JD (2012). Functional topography of the cerebellum for motor and cognitive tasks: an fMRI study. Neuroimage.

[32] Stoodley CJ (2012). The cerebellum and cognition: evidence from functional imaging studies. Cerebellum.

[33] Marien P (2014). Consensus paper: Language and the cerebellum: an ongoing enigma. Cerebellum.

[34] Baumann O (2015). Consensus paper: the role of the cerebellum in perceptual processes. Cerebellum.

[35] Sendhilnathan N, Goldberg ME, Ipata AE (2022). Mixed selectivity in the cerebellar Purkinje-cell response during visuomotor association learning. J. Neurosci..

[36] Lixenberg A, Yarkoni M, Botschko Y, Joshua M (2020). Encoding of eye movements explains reward-related activity in cerebellar simple spikes. J. Neurophysiol..

[37] Sendhilnathan N, Ipata A, Goldberg ME (2021). Mid-lateral cerebellar complex spikes encode multiple independent reward-related signals during reinforcement learning. Nat. Commun..

[38] Picard N, Strick PL (2001). Imaging the premotor areas. Curr. Opin. Neurobiol..

[39] Sendhilnathan N, Ipata AE, Goldberg ME (2020). Neural correlates of reinforcement learning in midlateral cerebellum. Neuron.

[40] Attwell PJ, Cooke SF, Yeo CH (2002). Cerebellar function in consolidation of a motor memory. Neuron.

[41] Fischer B, Boch R, Ramsperger E (1984). Express-saccades of the monkey: effect of daily training on probability of occurrence and reaction time. Exp. Brain Res..

[42] Snider RS, Eldred E (1952). Cerebro-cerebellar relationships in the monkey. J. Neurophysiol..

[43] Bostan AC, Dum RP, Strick PL (2010). The basal ganglia communicate with the cerebellum. Proc. Natl Acad. Sci. USA.

[44] Voogd J, Glickstein M (1998). The anatomy of the cerebellum. Trends Neurosci..

[45] Brodal P, Brodal A (1981). The olivocerebellar projection in the monkey. Experimental studies with the method of retrograde tracing of horseradish peroxidase. J. Comp. Neurol..

[46] Lu MT, Preston JB, Strick PL (1994). Interconnections between the prefrontal cortex and the premotor areas in the frontal lobe. J. Comp. Neurol..

[47] Luppino G, Rozzi S, Calzavara R, Matelli M (2003). Prefrontal and agranular cingulate projections to the dorsal premotor areas F2 and F7 in the macaque monkey. Eur. J. Neurosci..

[48] Barbas H, Pandya DN (1987). Architecture and frontal cortical connections of the premotor cortex (area 6) in the rhesus monkey. J. Comp. Neurol..

[49] Raymond JL (2020). Research on the cerebellum yields rewards. Nature.

[50] Wise SP, Murray EA (2000). Arbitrary associations between antecedents and actions. Trends Neurosci..

[51] He SQ, Dum RP, Strick PL (1993). Topographic organization of corticospinal projections from the frontal lobe: motor areas on the lateral surface of the hemisphere. J. Neurosci..

[52] Grafton ST, Fagg AH, Arbib MA (1998). Dorsal premotor cortex and conditional movement selection: a PET functional mapping study. J. Neurophysiol..

[53] Nakayama Y, Yamagata T, Hoshi E (2016). Rostrocaudal functional gradient among the pre-dorsal premotor cortex, dorsal premotor cortex and primary motor cortex in goal-directed motor behaviour. Eur. J. Neurosci..

[54] Boussaoud D (2001). Attention versus intention in the primate premotor cortex. Neuroimage.

[55] Abe M, Hanakawa T (2009). Functional coupling underlying motor and cognitive functions of the dorsal premotor cortex. Behav. Brain Res..

[56] Petrides M (1982). Motor conditional associative-learning after selective prefrontal lesions in the monkey. Behav. Brain Res..

[57] Fiez JA, Petersen SE, Cheney MK, Raichle ME (1992). Impaired non-motor learning and error detection associated with cerebellar damage. A single case study. Brain.

[58] Tucker J (1996). Associative learning in patients with cerebellar ataxia. Behav. Neurosci..

[59] Toni I, Rushworth MF, Passingham RE (2001). Neural correlates of visuomotor associations. Spatial rules compared with arbitrary rules. Exp. Brain Res..

[60] Boettiger CA, D’Esposito M (2005). Frontal networks for learning and executing arbitrary stimulus-response associations. J. Neurosci..

[61] Madhavan R (2019). Neural interactions underlying visuomotor associations in the human brain. Cereb Cortex.

[62] Nixon PD, Passingham RE (2000). The cerebellum and cognition: cerebellar lesions impair sequence learning but not conditional visuomotor learning in monkeys. Neuropsychologia.

[63] Nixon PD, Passingham RE (1999). The cerebellum and cognition: cerebellar lesions do not impair spatial working memory or visual associative learning in monkeys. Eur. J. Neurosci..

[64] Mandolesi L, Leggio MG, Spirito F, Federico F, Petrosini L (2007). Is the cerebellum involved in the visuo-locomotor associative learning?. Behav. Brain Res..

[65] Stoodley CJ (2017). Altered cerebellar connectivity in autism and cerebellar-mediated rescue of autism-related behaviors in mice. Nat. Neurosci..

[66] Badura A (2018). Normal cognitive and social development require posterior cerebellar activity. Elife.

[67] Deverett B, Kislin M, Tank DW, Wang SS (2019). Cerebellar disruption impairs working memory during evidence accumulation. Nat. Commun..

[68] Proville RD (2014). Cerebellum involvement in cortical sensorimotor circuits for the control of voluntary movements. Nat. Neurosci..

[69] Kelly E (2020). Regulation of autism-relevant behaviors by cerebellar-prefrontal cortical circuits. Nat. Neurosci..

[70] Chabrol FP, Blot A, Mrsic-Flogel TD (2019). Cerebellar contribution to preparatory activity in motor neocortex. Neuron.

[71] Bina L, Romano V, Hoogland TM, Bosman LWJ, De Zeeuw CI (2022). Purkinje cells translate subjective salience into readiness to act and choice performance. Cell Rep..

[72] Suzuki L, Coulon P, Sabel-Goedknegt EH, Ruigrok TJ (2012). Organization of cerebral projections to identified cerebellar zones in the posterior cerebellum of the rat. J. Neurosci..

[73] Habas C (2009). Distinct cerebellar contributions to intrinsic connectivity networks. J. Neurosci..

[74] Krienen FM, Buckner RL (2009). Segregated fronto-cerebellar circuits revealed by intrinsic functional connectivity. Cereb. Cortex.

[75] O’Reilly JX, Beckmann CF, Tomassini V, Ramnani N, Johansen-Berg H (2010). Distinct and overlapping functional zones in the cerebellum defined by resting state functional connectivity. Cereb. Cortex.

[76] Guell X, Schmahmann JD, Gabrieli J, Ghosh SS (2018). Functional gradients of the cerebellum. Elife.

[77] Balsters JH, Zerbi V, Sallet J, Wenderoth N, Mars RB (2020). Primate homologs of mouse cortico-striatal circuits. Elife.

[78] Histed MH, Pasupathy A, Miller EK (2009). Learning substrates in the primate prefrontal cortex and striatum: sustained activity related to successful actions. Neuron.

[79] Glickstein M, May JG, Mercier BE (1985). Corticopontine projection in the macaque: the distribution of labelled cortical cells after large injections of horseradish peroxidase in the pontine nuclei. J. Comp. Neurol..

[80] De Zeeuw CI (1998). Microcircuitry and function of the inferior olive. Trends Neurosci..

[81] Wagner MJ, Kim T, Savall J, Schnitzer MJ, Luo L (2017). Cerebellar granule cells encode the expectation of reward. Nature.

[82] Kostadinov D, Beau M, Pozo M, Häusser M (2019). Predictive and reactive reward signals conveyed by climbing fiber inputs to cerebellar Purkinje cells. Nat. Neurosci..

[83] Heffley W, Hull C (2019). Classical conditioning drives learned reward prediction signals in climbing fibers across the lateral cerebellum. eLife.

[84] Heffley W (2018). Coordinated cerebellar climbing fiber activity signals learned sensorimotor predictions. Nat. Neurosci..

[85] Ohmae S, Medina JF (2015). Climbing fibers encode a temporal-difference prediction error during cerebellar learning in mice. Nat. Neurosci..

[86] Person, A. L. & Raman, I. M. Purkinje neuron synchrony elicits time-locked spiking in the cerebellar nuclei. *Nature* **481**, 502–505 (2012). 10.1038/nature10732 PMC3268051 22198670

[87] Hoshi E, Tremblay L, Feger J, Carras PL, Strick PL (2005). The cerebellum communicates with the basal ganglia. Nat. Neurosci..

[88] Bussey TJ, Wise SP, Murray EA (2001). The role of ventral and orbital prefrontal cortex in conditional visuomotor learning and strategy use in rhesus monkeys (Macaca mulatta). Behav. Neurosci..

[89] Puig VM, Miller EK (2012). The role of prefrontal dopamine D1 receptors in the neural mechanisms of associative learning. Neuron.

[90] Bostan AC, Strick PL (2018). The basal ganglia and the cerebellum: nodes in an integrated network. Nat. Rev. Neurosci..

[91] Diedrichsen J, King M, Hernandez-Castillo C, Sereno M, Ivry RB (2019). Universal transform or multiple functionality? Understanding the contribution of the human cerebellum across task domains. Neuron.

[92] Schmahmann JD (1996). From movement to thought: anatomic substrates of the cerebellar contribution to cognitive processing. Hum. Brain Mapp..

[93] De Zeeuw CI, Lisberger SG, Raymond JL (2021). Diversity and dynamism in the cerebellum. Nat. Neurosci..

[94] Sugihara I, Shinoda Y (2004). Molecular, topographic, and functional organization of the cerebellar cortex: a study with combined aldolase C and olivocerebellar labeling. J. Neurosci..

[95] Pimenta AF, Strick PL, Levitt P (2001). Novel proteoglycan epitope expressed in functionally discrete patterns in primate cortical and subcortical regions. J. Comp. Neurol..

[96] Kebschull JM (2020). Cerebellar nuclei evolved by repeatedly duplicating a conserved cell-type set. Science.

[97] Rieubland S, Roth A, Hausser M (2014). Structured connectivity in cerebellar inhibitory networks. Neuron.

[98] Arlt C, Hausser M (2020). Microcircuit rules governing impact of single interneurons on purkinje cell output in vivo. Cell Rep.

[99] Herzfeld DJ, Kojima Y, Soetedjo R, Shadmehr R (2015). Encoding of action by the Purkinje cells of the cerebellum. Nature.

[100] Herzfeld DJ, Kojima Y, Soetedjo R, Shadmehr R (2018). Encoding of error and learning to correct that error by the Purkinje cells of the cerebellum. Nat. Neurosci..

[101] Medina JF, Lisberger SG (2008). Links from complex spikes to local plasticity and motor learning in the cerebellum of awake-behaving monkeys. Nat. Neurosci..

[102] Larry N, Yarkoni M, Lixenberg A, Joshua M (2019). Cerebellar climbing fibers encode expected reward size. Elife.

[103] Popa LS, Hewitt AL, Ebner TJ (2013). Purkinje cell simple spike discharge encodes error signals consistent with a forward internal model. Cerebellum.

[104] Popa LS, Streng ML, Hewitt AL, Ebner TJ (2016). The errors of our ways: understanding error representations in cerebellar-dependent motor learning. Cerebellum.

[105] Ma M (2020). Molecular layer interneurons in the cerebellum encode for valence in associative learning. Nat. Commun..

[106] Kelly RM, Strick PL (2000). Rabies as a transneuronal tracer of circuits in the central nervous system. J Neurosci. Methods.

[107] Hays Jr, A., Richmond, B. & Optican, L. *Unix-based Multiple-process System, for Real-time Data Acquisition and Control*. In: WESCON Proceedings Conference, 1-10 (1982).

[108] Larsell O (1953). The cerebellum of the cat and the monkey. J. Comp. Neurol..

[109] Liu Y, Yttri EA, Snyder LH (2010). Intention and attention: different functional roles for LIPd and LIPv. Nat Neurosci..

[110] Arikan R (2002). A method to measure the effective spread of focally injected muscimol into the central nervous system with electrophysiology and light microscopy. J. Neurosci. Methods.

[111] Tinevez J-YY (2017). TrackMate: An open and extensible platform for single-particle tracking. Methods.

[112] Schindelin J (2012). Fiji: an open-source platform for biological-image analysis. Nat. Methods.

[113] Greger B, Norris SA, Thach WT (2004). Spike firing in the lateral cerebellar cortex correlated with movement and motor parameters irrespective of the effector limb. J. Neurophysiol..

[114] Sendhilnathan N, Basu D, Goldberg ME, Schall JD, Murthy A (2021). Neural correlates of goal-directed and non–goal-directed movements. Proc. Natl Acad. Sci. USA.

[115] Sendhilnathan N, Basu D, Murthy A (2017). Simultaneous analysis of the LFP and spiking activity reveals essential components of a visuomotor transformation in the frontal eye field. Proc. Natl Acad. Sci. USA.

[116] Basu D, Sendhilnathan N, Murthy A (2021). Neural mechanisms underlying the temporal control of sequential saccade planning in the frontal eye field. Proc. Natl Acad. Sci. USA.

[117] Sendhilnathan N, Basu D, Murthy A (2020). Assessing within‐trial and across‐trial neural variability in macaque frontal eye fields and their relation to behaviour. Eur. J. Neurosci..

[118] Nadin-Davis SA (2000). A panel of monoclonal antibodies targeting the rabies virus phosphoprotein identifies a highly variable epitope of value for sensitive strain discrimination. J. Clin. Microbiol..

