# Supplementary material for: A cerebro-cerebellar network for learning visuomotor associations
Source: Nat Commun. 2024 Mar 21;15:2519. doi: 10.1038/s41467-024-46281-0 (PMC10957870; doi:10.1038/s41467-024-46281-0)
Supplement: Supplementary file 1 — Supplementary Information [file 41467_2024_46281_MOESM1_ESM.docx]

**SUPPLEMENTARY NOTES:**

**Supplementary Note 1: A computational framework for learning visuomotor associations:**

Our results indicate that the lateral posterior cerebellum is connected with the prefrontal cortex. Because inactivation of the lateral posterior cerebellum significantly impairs the ability of monkeys to learn new visuomotor associations, we expect that the role of the cerebellum may be to provide the reinforcement learning error signal necessary for learning this mental skill, in a distributed brain-wide network. As a first approximation of this network function, we modeled the interaction between the cerebellum and the prefrontal cortex by combing the functions of both these brain areas. We used a computational model which combined a Q-reinforcement learning model, implemented by the lateral posterior cerebellum^1^, and a decision-making model based on choice discrimination^2^, implemented by the prefrontal cortex ^3^.

Consider learning one symbol-choice-reward association between a given symbol and say, left hand manipulandum release. During such a choice discrimination, neurons in prefrontal cortex increase their firing rate by accumulating evidence with a certain rate ^3^. Some prefrontal cortical neurons accumulate evidence for the left choice, and some for the right choice. That is, there is a race to threshold for these accumulators and through a winner-takes-all mechanism, the choice that was most popular will be executed. In our example, during overtrained trials (OT), the rate of accumulation for the left choice is much higher than the right choice, leading to the left choice execution with a faster reaction time. However, at the beginning of learning, the rates of both accumulators are equivalent, and lower than the OT level, since evidence accumulation takes longer during uncertainty^4^, as reflected in an increase in reaction time during early learning (**Fig 2g-i**). Therefore, the choice that is executed on a given trial during early learning will be randomly chosen, depending on whichever accumulator reaches the threshold first, by accumulating evidence randomly. The outcome of this decision can be transferred to interconnected regions of the cerebellar cortex, where it is represented by Purkinje cell (P-cell) simple spike activity, until the next decision is made ^1^ (**Fig S3a**). More importantly, the magnitude of the outcome represents the amount of “unfamiliarity” of the association, or the amount of learning that needs to happen. In other words, during early learning, the magnitude of this error signal is high, as represented by the Q value in the classic reinforcement learning model^5^. The output Q value from the cerebellum can be used as an input to interconnected regions in the prefrontal cortex to control the rate of accumulation of the neurons during choice discrimination (**Fig S3a-b**). During learning, cerebellar P-cells modulate the accumulation rate of cortical neurons, and cortical neurons modulate the error magnitude of P-cells. Consequently, the rate of the left choice accumulator keeps decreasing, and the rate of the right choice accumulator increases slightly from trial to trial (**Fig S3c**). At the end of learning, the rate of the left choice accumulator approaches that of the OT values, thus evoking faster reaction times. This results in a gradual decrease of reaction time from the start to the end of learning (**Fig 2g-i**). This model successfully predicts the monkey’s choice behavior, learning strategy, and the rate of learning, given the P-cell simple spike activity and the reaction time (through which the rates of accumulators are estimated, a proxy for prefrontal cortical activity) as the inputs ^1^.

During muscimol inactivation, we modeled the P-cell activity as Gaussian white noise, since the P-cell activity is eliminated. The absence of P-cell activity does not majorly impact the rate of accumulation in the OT task and thus, the process looks similar to the control condition. Behaviorally, the performance (**Fig 1j**) and the reaction time (**Fig 2g-i**) during muscimol inactivation would be comparable to the saline condition during OT. However, through learning, the rate of accumulation in the prefrontal cortex cannot be controlled or optimized by the cerebellar output, leading to noisy choice discrimination and an impairment in learning. That is, the rate of accumulators cannot be changed as efficiently as before. This could lead to impairment in performance (in **Fig 1f-i**) and no significant change in reaction time (**Fig 2g-i**), as predicted by our model (**Fig S3d**). The eventual change might be brought forth by the influence of other centers of the brain, such as the basal ganglia (**Fig S16**). Alternatively, because both cerebellar hemispheres are active in the task for both hands, the prefrontal cortex might receive cerebellar information via cortico-cortical connections between the two hemispheres. Here, we have focused on the main contralateral projection from the cerebellum to the prefrontal cortex, but ipsilateral projections are also present and likely play a role in task performance.

**Supplementary Note 2: Learning vs consolidation during reinforcement learning:**

Inactivation of the lateral posterior region inhibits learning new visuomotor associations, but has no effect well-learned associations or on movement kinematics. A similar dissociation between acquisition and consolidation has been demonstrated in the vestibulo-ocular reflex during motor learning ^6^. This suggests that the locus of consolidation of learning is different from locus of acquisition (cerebellar cortex). In the case of the vestibulo-ocular reflex, this extra-cerebellar locus is the medial vestibular nucleus^7^. In the case of reinforcement learning, the synaptic changes associated with learning that begin in the cerebellum might migrate to interconnected regions, such as the prefrontal cortex, the basal ganglia, or both.

**Supplementary References:**

1 Sendhilnathan, N., Ipata, A. E. & Goldberg, M. E. Neural correlates of reinforcement learning in midlateral cerebellum. *Neuron* **106** (2020).

2 Ratcliff, R. & McKoon, G. The diffusion decision model: theory and data for two-choice decision tasks. *Neural computation* **20**, 873-922 (2008).

3 Kim, J.-N. & Shadlen, M. N. Neural correlates of a decision in the dorsolateral prefrontal cortex of the macaque. *Nature neuroscience* **2**, 176-185 (1999).

4 Kiani, R., Corthell, L. & Shadlen, M. N. Choice certainty is informed by both evidence and decision time. *Neuron* **84**, 1329-1342 (2014).

5 Sutton, R. S. & Barto, A. G. *Introduction to reinforcement learning*. Vol. 135 (MIT press Cambridge, 1998).

6 Kassardjian, C. D. *et al.* The site of a motor memory shifts with consolidation. *Journal of Neuroscience* **25**, 7979-7985 (2005).

7 Lisberger, S. G. Cerebellar LTD: a molecular mechanism of behavioral learning? *Cell* **92**, 701-704 (1998).

8 Brodal, P. & Brodal, A. The olivocerebellar projection in the monkey. Experimental studies with the method of retrograde tracing of horseradish peroxidase. *J Comp Neurol* **201**, 375-393 (1981).

9 Haynes, W. I. & Haber, S. N. The organization of prefrontal-subthalamic inputs in primates provides an anatomical substrate for both functional specificity and integration: implications for Basal Ganglia models and deep brain stimulation. *J Neurosci* **33**, 4804-4814 (2013).

10 Calzavara, R., Mailly, P. & Haber, S. N. Relationship between the corticostriatal terminals from areas 9 and 46, and those from area 8A, dorsal and rostral premotor cortex and area 24c: an anatomical substrate for cognition to action. *Eur J Neurosci* **26**, 2005-2024 (2007).

11 Bostan, A. C., Dum, R. P. & Strick, P. L. The basal ganglia communicate with the cerebellum. *Proc Natl Acad Sci U S A* **107**, 8452-8456 (2010).

12 Hoshi, E., Tremblay, L., Feger, J., Carras, P. L. & Strick, P. L. The cerebellum communicates with the basal ganglia. *Nat Neurosci* **8**, 1491-1493 (2005).

13 Middleton, F. A. & Strick, P. L. Basal ganglia output and cognition: evidence from anatomical, behavioral, and clinical studies. *Brain Cogn* **42**, 183-200 (2000).

**
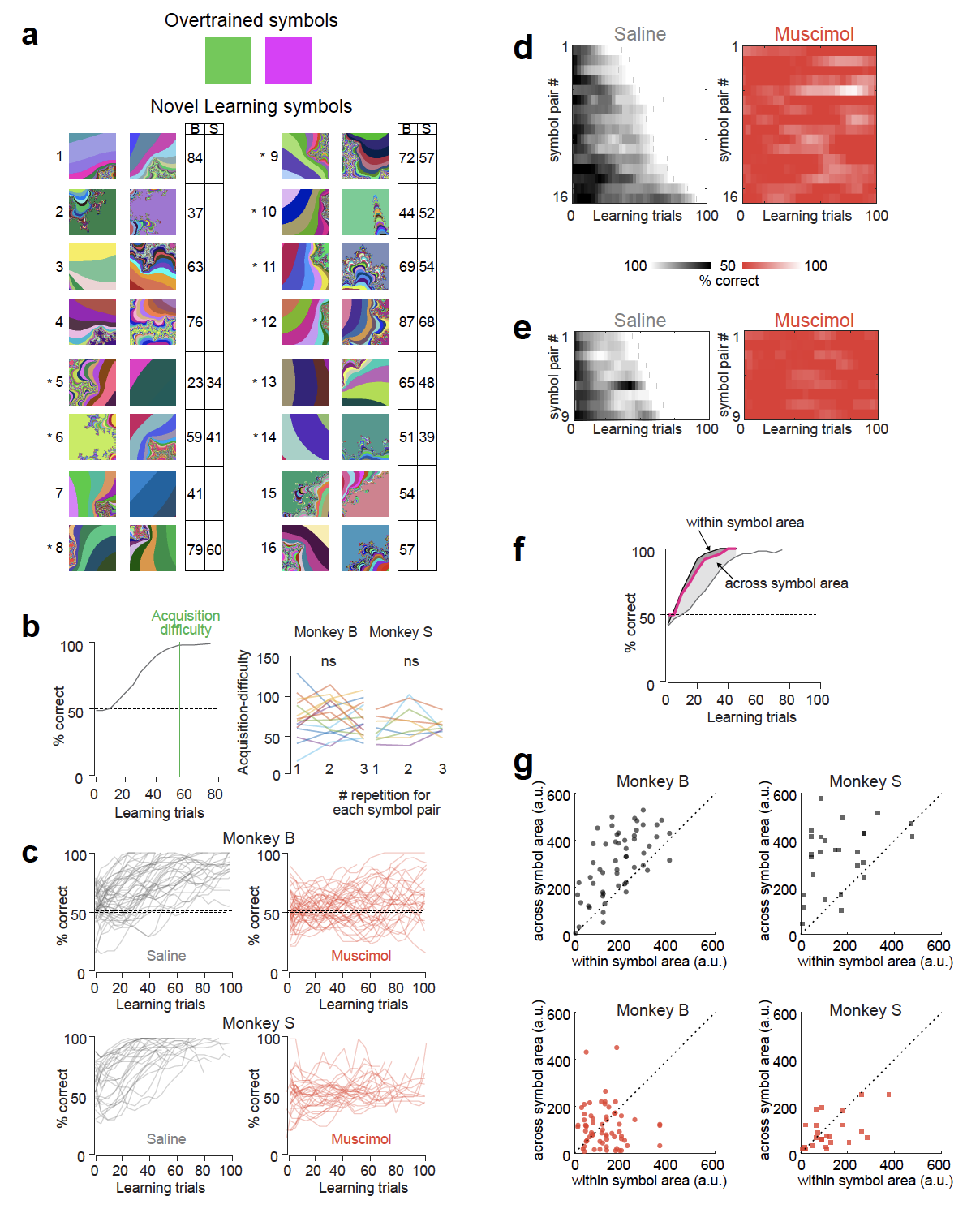
**

**Figure S1: Library of symbols and behavioral metrics**

1. Overtrained symbols are shown on the top followed by 16 pairs of novel symbols. All these 16 pairs were used for monkey B. Only the 9 pairs marked by * were randomly chosen to be used for monkey S. The numbers to the right indicate the acquisition difficulty rate for monkey B (first column) and monkey S (second column).
2. Left: A schematic illustration of the acquisition-difficulty (trial number at 90% correct). Right: Acquisition-difficulty for each symbol pair (colored lines) per repetition, for both monkeys. Acquisition-difficulty did not change with repetition number of the same symbol pair (monkey B: P = 0.46; monkey S: P = 0.63, ANOVA).
3. Same as Fig 1F(top left) and 1G(top right) for monkey B and monkey S (bottom).
4. Left: heat plot of learning curves (each row) for the behavioral performaces of 16 pairs of symbols, arranged in the increasing oder of acquisation difficulty during the saline-control condition. Grey tick marks indicate the trial at which recording was stopped. Right: heat plot of learning curves (each row) for the behavioral performaces of the same16 pairs of symbols (in the same oder of left panel) during cerebellar inactivation. The color scale is shown below the panel.
5. Same as **e**, but for monkey S.
6. Schematic of the analysis method: consider a randomly chosen ‘reference’ learning curve for a randomly chosen symbol pair (thick pink line). The dark gray shaded region represents the area between this reference learning curve and another randomly chosen learning curve for the same symbol (within-symbol area). The light gray shaded region represents the area between the reference learning curve and another randomly chosen learning curve from a different symbol (across-symbol area). To compare both these areas, they were normalized by the length of the shortest of the three curves. This analysis was done by choosing each session’s data as the reference.
7. Top: quantitation of learning curves during saline condition analyzed as shown in **Fig S1f** for monkey B (left) and monkey S (right). P<0.001; paired t-test, for both monkeys. Bottom: Same as top but for muscimol condition. P = 0.81 paired t-test for Monkey B and P = 0.43 paired t-test for monkey S.

Data is shown as mean ± SEM.


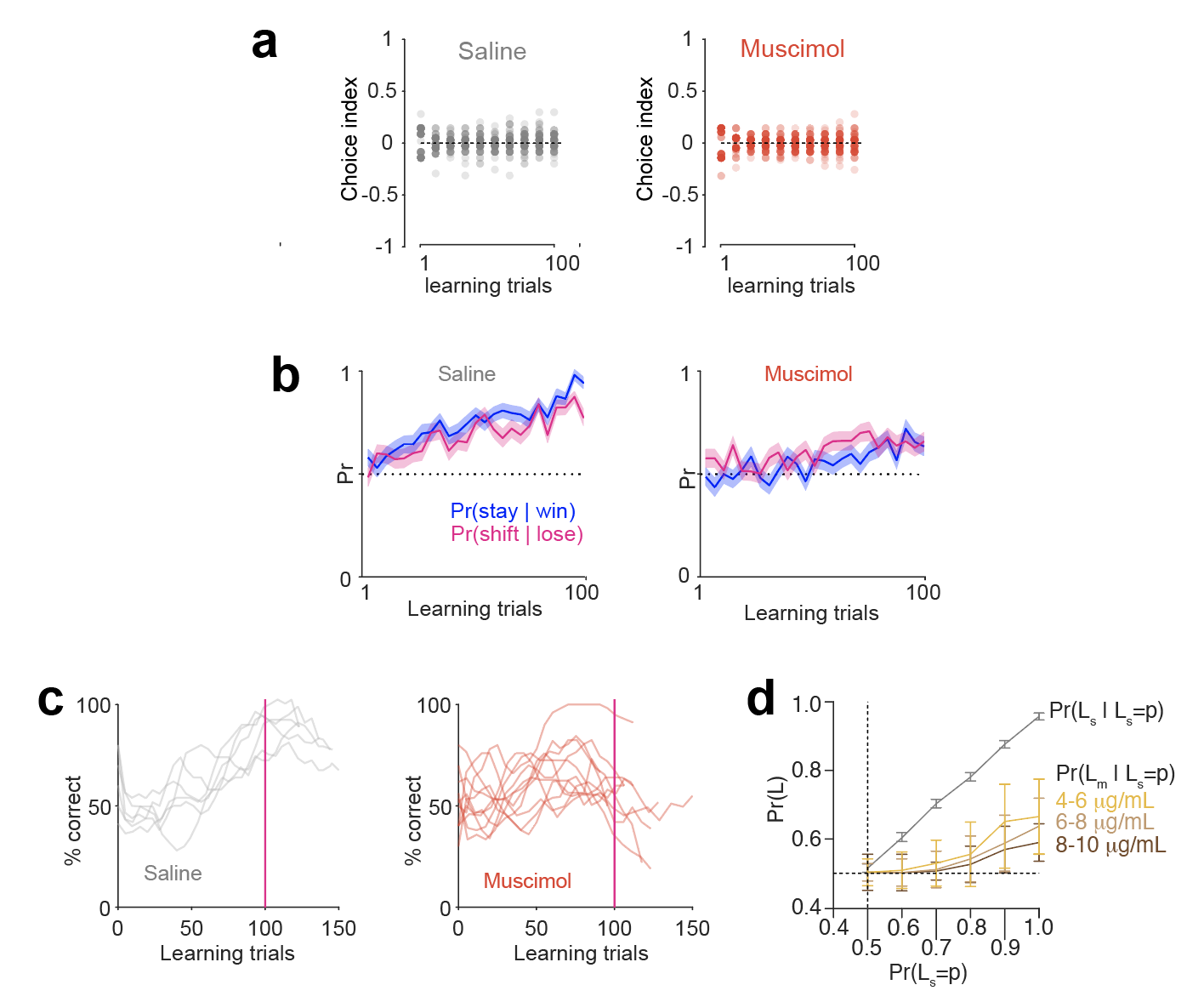


**Figure S2: Effect of different treatments on behavior and learning**

1. Choice index for the saline and muscimol conditions. The choice index was calculated as (L-R/L+R) where L is the number of left choices and R is the number of right choices, in a moving window of 10 trials shifted by 10 trials for control (top) and inactivation (bottom) sessions. Values close to 0 indicate a lack of choice bias and values close to ±1 indicate strong choice bias.
2. Probabilities of win-stay and lose-switch during learning from both monkeys during saline-control (left) and cerebellar inactivation conditions (right).
3. Mean reward prediction error as a function of learning for saline (gray) and muscimol (red) conditions (see **Methods**). Inset: learning rates estimated from delta learning model fitting.
4. Learning curves for sessions with more than 100 trials. The behavioral performance in saline and muscimol conditions tend to decline roughly after 100 trials (marked by pink vertical line).
5. Effect of different muscimol concentrations on behavior. Same format as **Fig 1i**.

Data is shown as mean ± SEM.

**
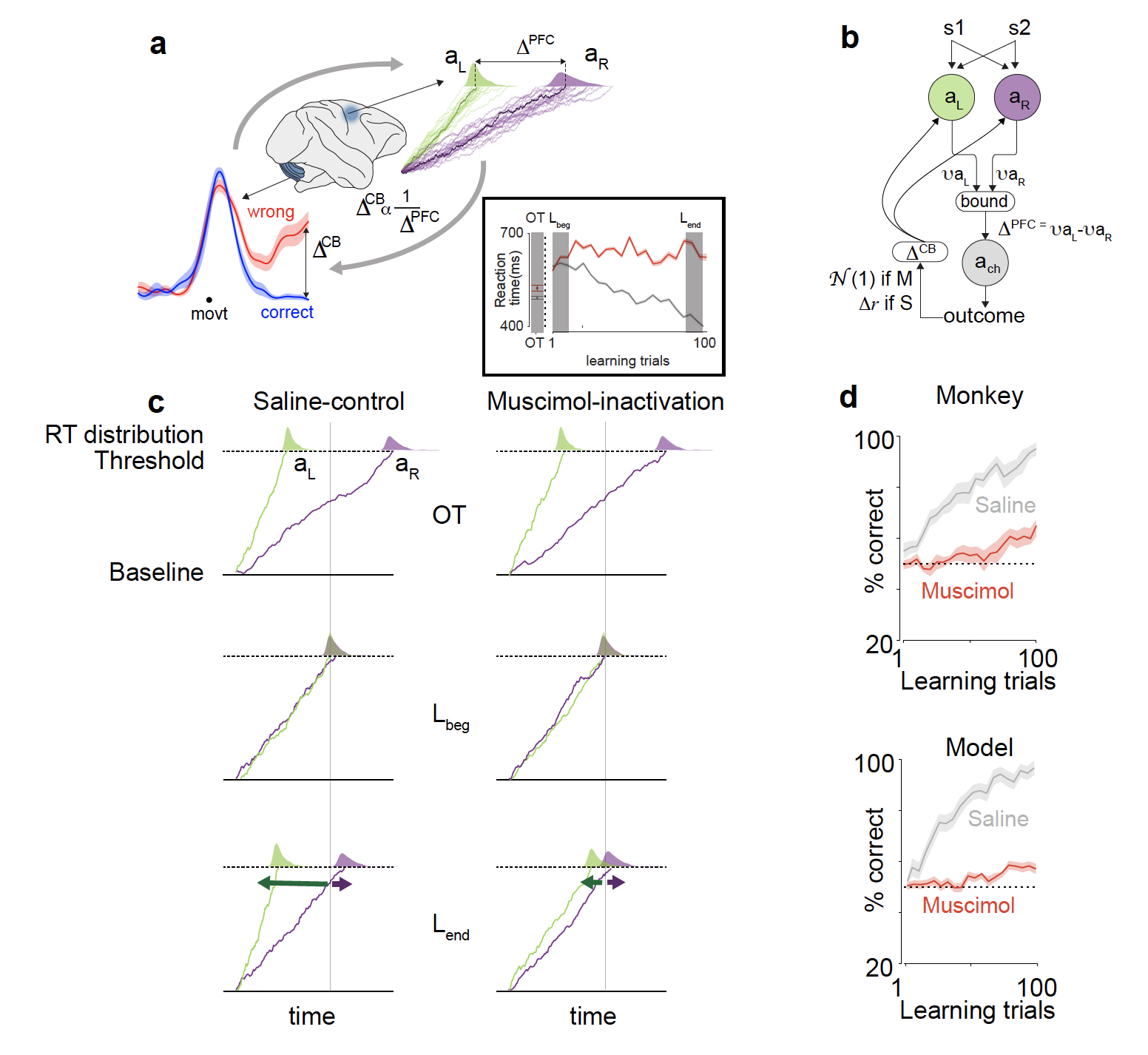
**

**Figure S3: A computational framework involving the cerebellum for learning visuomotor association**

1. Schematic illustration of the computational model. The primate brain is shown in the center highlighting the posterior lateral cerebellum on the bottom left and prePMd on the top right. A representative P-cell showing reinforcement error signal as the difference between the neural activities for wrong (red) and correct (blue) trials^1^ is shown in the bottom left. A race to threshold accumulator framework implemented by the cortical neurons is shown on the top right, for left (green) and right (purple) manipulanda release choices. Inset: Same as **Fig 3g** but with three phases marked in gray (OT, L_beg_, L_end_) that correspond to corresponding panels in **Fig S3c** below.
2. Top: Reinforcement-drift diffusion model. Action choices are modeled as accumulators with rates υ_aL_ and υ_aR_, racing to threshold (bound). The winner takes all and consequences of the chosen action a_ch_ is evaluated by the activity of P-cells given by Δ^CB^. This is used to update the rates of the accumulator on a trial by trial basis. For saline-control condition, Δ ^CB^ is given by the difference in firing rate of correct and wrong outcome in the delta epoch of the P-cells^1^. For muscimol condition, this is a random number drawn from a Gaussian distribution.
3. A schematic illustration of the working of the model described in **Fig S3c.** Left panel shows the saline-control condition for OT (top), beginning of learning (middle) and end of learning (bottom). The two example accumulators for left (green; a_L_) and right (purple; a_R_) choices for one representative trial are shown in each case, with the reaction distributions on the top. Right panel shows the accumulator dynamics in the muscimol-inactivation condition. These are shown for three learning phases illustrated in **Fig S3a** inset.
4. Learning curve for saline and muscimol conditions from monkey (top) and model predictions (bottom).

Data is shown as mean ± SEM.

**
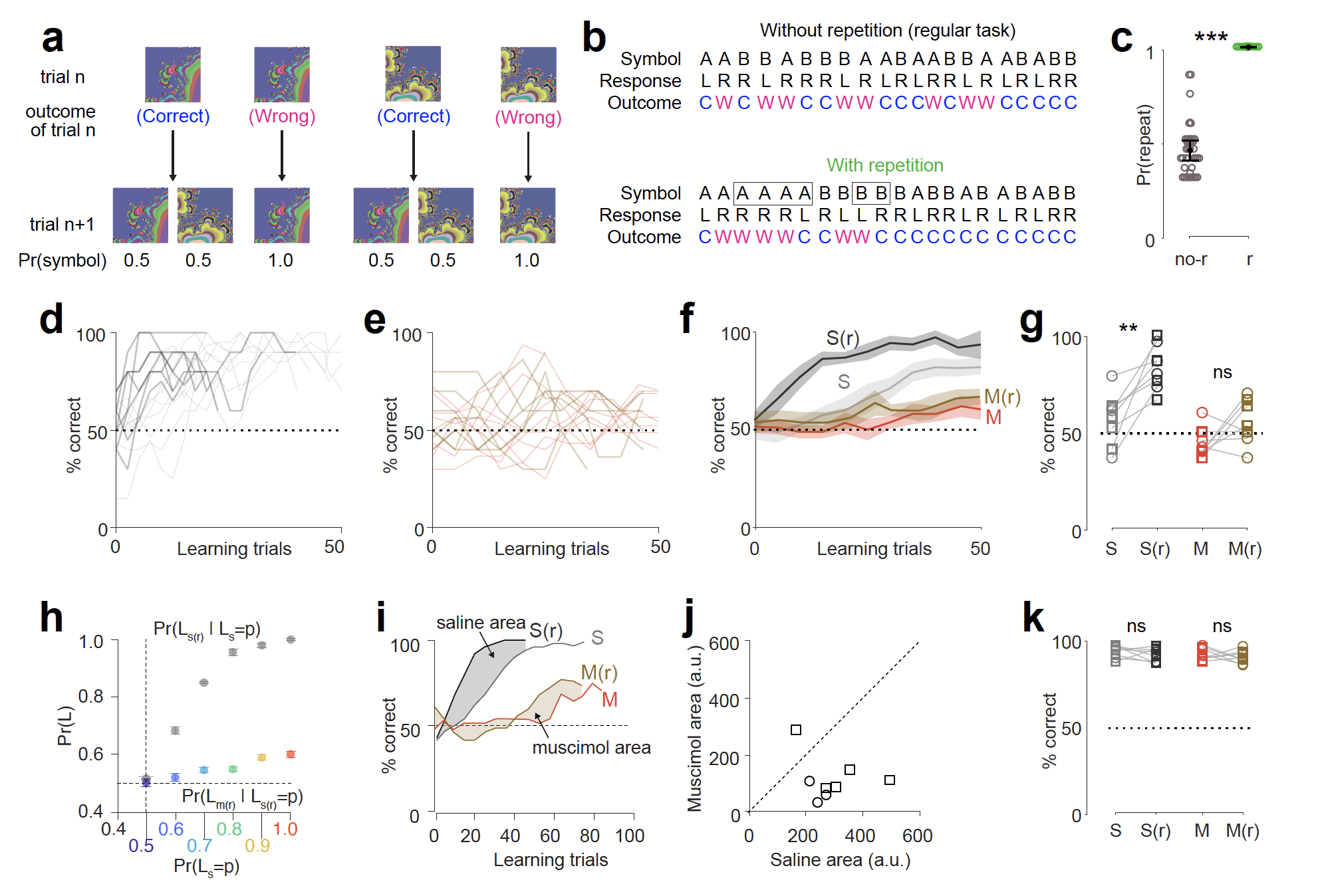
**

**Figure S4: Repetition of symbols after error trials during learning significantly improved the performance during control-saline condition but not during lateral-posterior cerebellar inactivation**

1. Task paradigm for repetition of symbols: If the monkey got a trial wrong, the same symbol was presented again in the next trial; but if the monkey got the trial right, one of the two symbols was presented with equal probabilities in the next trial.
2. An example of stimulus-response-outcome sequence for the task without symbol repetition (top) and with symbol repetition (bottom). A and B indicate two symbols, L and R indicate left and right responses; C and W indicate correct and wrong outcomes. Note that if a trial was wrong in the symbol repetition paradigm, the same symbol was repeated in the subsequent trials (marked by a rectangle) until the monkey made a correct response.
3. Beeswarm plot of the probability of repetition of the same symbol after a wrong trial, in the normal task (left) and the repeat task (right). ***P <0.001; Mann-Whitney U test.
4. Behavioral performance for both monkeys for each session during novel learning task for control-saline with repetition (dark gay) and without repetition (light gray) conditions.
5. Same as **d** but for lateral-posterior cerebellar inactivation condition with repetition (brown) and without repitition (red) conditions.
6. Mean learning curve (from both monkeys) for saline-control (S), saline-control with repeat (S(r)), muscimol (M) and muscimol with repeat (M(r)) conditions.
7. Quantitation from **c** in the trial window of 10-20 trials, for individual sessions, for two monkeys separately (circles are Monkey B; squares are Monkey S). Repetition of symbols during learning significantly improved the performance (rate of learning) during control-saline condition (Monkey B: P<0.05, paired t-test; Monkey S: P<0.05, paired t-test) but not during cerebellar inactivation (Monkey B: P = 0.69; paired t-test; Monkey S: P = 0.09, paired t-test).
8. Same analysis as **Fig 1h-i** but for data in the repetition experiment. Gray markers represent Pr(L_S(r)_ | L_S_ = p) where S(r) represents saline with repetition condition and S represents saline without repetition condition. The colored markers represent Pr(L_M(r)_ | L_s(r)_ = p) where M(r) represents muscimol with repetition condition and m represents muscimol without repetition condition. Same format as **Fig 1i**.
9. Shematic of the analysis method: The gray shaded region represents the area between two randomly chosen learning curves for the same symbol under saline condition– one from without repetition condition and another with repetition condition (saline area). The brown shaded region represents the area between two randomly chosen learning curves for the same symbol under mussimol condition– one from without repetition condition and another with repetition condition (muscimol area). To compare both these areas, they were normalized by the length of the shortest of the four curves.
10. Quantitation of learning curves as shown in **Fig S4i** for both monkeys.
11. Quantitation of % correct in the overtrained task for individual sessions, for two monkeys separately (circles are Monkey B; squares are Monkey S). Repetition of symbols during learning did not change the performance in the overtrained task for saline or muscimol infusions.

Data is shown as mean ± SEM.

**
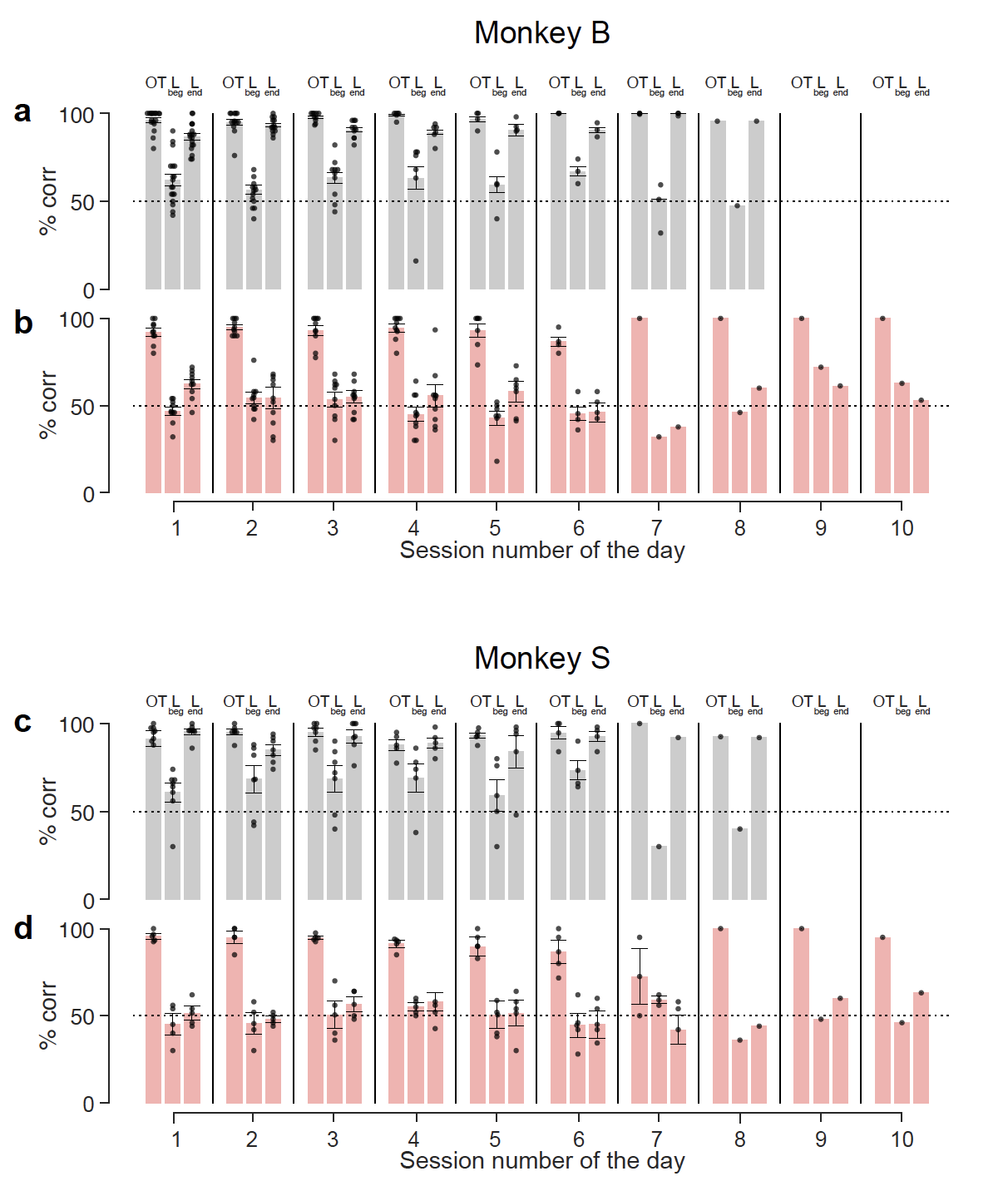
**

**Figure S5: Performance for different learning sessions, within days**

1. Behavioral performance for monkey B during saline-control condition for each session within days, for overtrained (OT), beginning of learning (L_beg_) and end of learning (L_end_).
2. Same as **Fig S5a**, but during lateral-posterior cerebellar inactivation condition.
3. Same as **Fig S5a**, but for monkey S.
4. Same as **Fig S5b,** but for monkey S.

Data is shown as mean ± SEM.

**
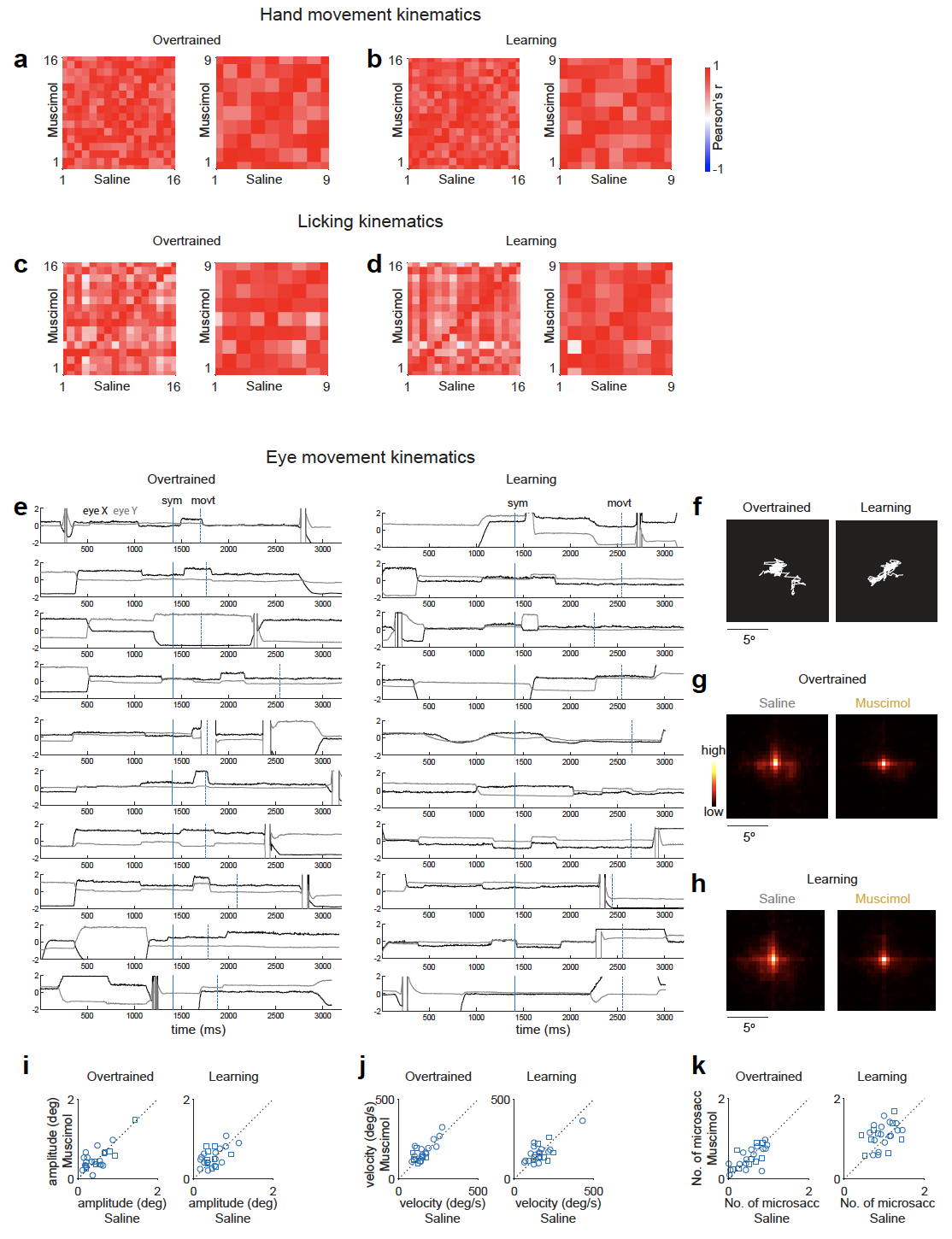
**

**Figure S6: Effect of different treatments on the hand, licking and eye motor kinematics**

1. Correlation matrix showing Pearsons’ r value between the hand movement trajectories in saline and muscimol conditions for each symbol pair in the overtrained task for monkey B (left) and monkey S (right). See inset for scale.
2. Same as **a** but for learning task.
3. Same as **a**, but for licking behavior.
4. Same as **b**, but for licking behavior.
5. Ten consecutive example trials showing raw x and y eye positions as a function of time for the same symbol (green square) in the overtrained and one of the fractal symbols in the learning task in the same session from the same monkey.
6. X and y eye positions from one example trial in the overtrained task (left) and learning (right) plotted against each other to show the eye movement made by the monkey during the trial.
7. Same as **f** but averaged across all trials in the overtrained task for saline (left) and muscimol (right) conditions across all sessions and both monkeys. See inset for scale.
8. Same as **g** but for learning task.
9. Saccade amplitudes in saline vs muscimol conditions for overtained task (left): Monkey B: P = 0.08 Wilcoxon signed-rank test (circle markers), Monkey S: P = 0.16 paired t-test (square markers); and learning task (right): Monkey B: P = 0.79 paired t-test (circle markers), Monkey S: P = 0.61 paired t-test (square markers).
10. Saccade velocity in saline vs muscimol conditions for overtained task (left): Monkey B: P = 0.78 Wilcoxon signed-rank test (circle markers), Monkey S: P = 0.49 Wilcoxon signed-rank test (square markers); and learning task (right): Monkey B: P = 0.28 Wilcoxon signed-rank test (circle markers), Monkey S: P = 0.44 Wilcoxon signed-rank test (square markers).
11. Number of miscrosaccades in saline vs muscimol conditions for overtained task (left): Monkey B: P = 0.34 Wilcoxon signed-rank test (circle markers), Monkey S: P = 0.68 Wilcoxon signed-rank test (square markers); and learning task (right): Monkey B: P = 0.78 Wilcoxon signed-rank test (circle markers), Monkey S: P = 0.23 Wilcoxon signed-rank test (square markers).

**
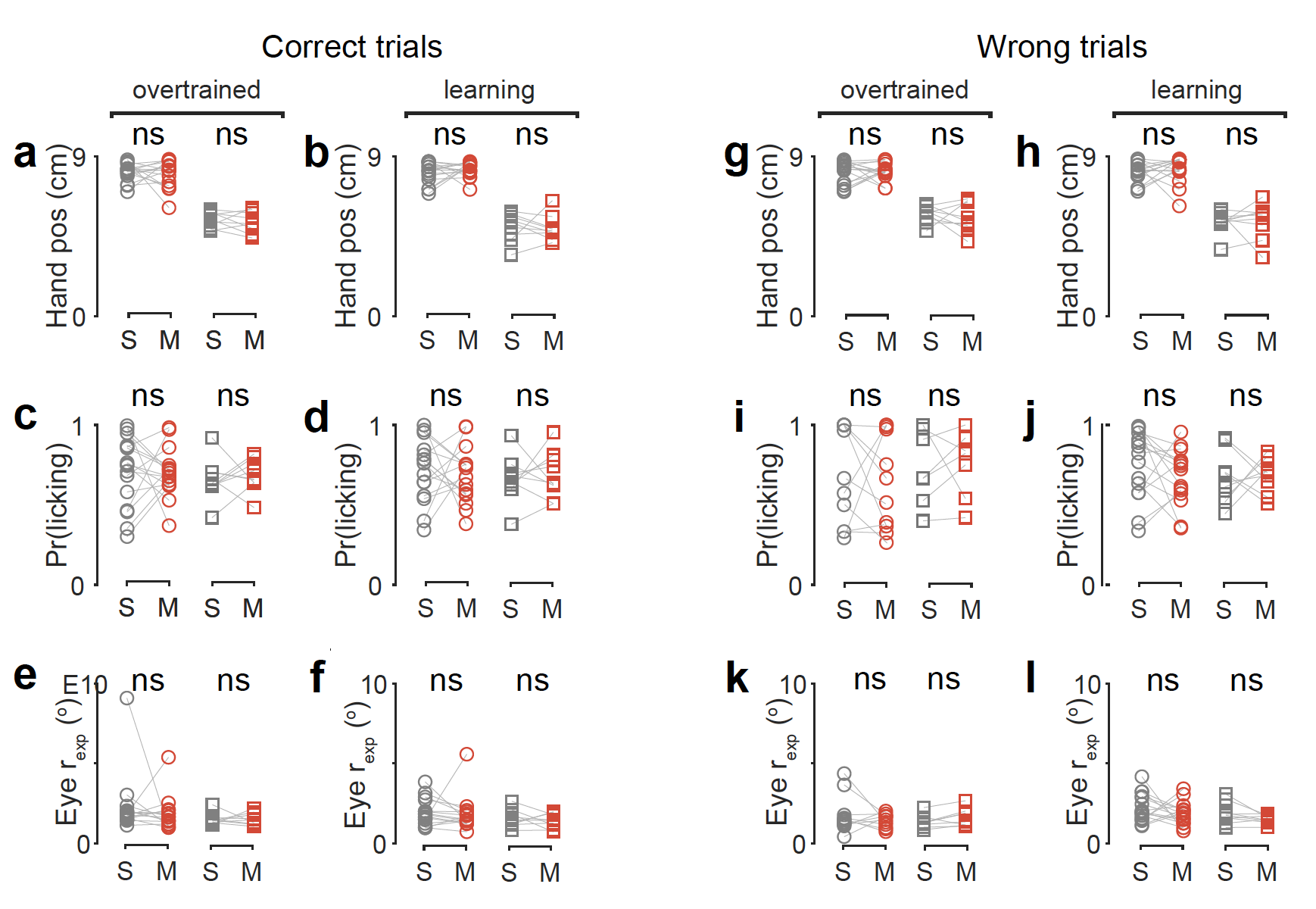
**

**Figure S7: Lateral-posterior cerebellar inactivation did not have differential effects on motor parameters for correct and wrong trials during learning**

1. Amplitude of hand position on correct trials, overtrained, Monkey B (left): P = 0.98; Mann-Whitney U test; monkey S (right), P = 0.54; paired t-test, N=25.
2. Amplitude of hand position on correct trials, learning, Monkey B (left): P = 0.46; paired t-test; Monkey S (right): P = 0.53; Mann-Whitney U test, N=25.
3. Probability of licking on correct trials, overtrained, Monkey B (left): P = 0.92; paired t-test; Monkey S (right): P = 0.63; paired t-test, N=25.
4. Probability of licking on correct trials, learning, Monkey B (left): P = 0.66; t-test; Monkey S (right): P = 0.73; paired t-test, N=25.
5. Visual exploration on correct trials, overtrained, Monkey B: P = 0.95; Mann-Whitney U test; Monkey S: P = 0.99; Mann-Whitney U test, N=25.
6. Visual exploration on correct trials, learning, Monkey B: P = 0.91; Mann-Whitney U test; Monkey S: P = 0.46; paired t-test, N=25.
7. Hand position on wrong trials, overtrained, Monkey B (left): P = 0.65; Mann-Whitney U test; Monkey S (right): P = 0.58; paired t-test, N=25.
8. Hand position on wrong trials, learning, Monkey B (left): P = 0.20; ranskum test; Monkey S (right): P = 0.59; Mann-Whitney U test, N=25.
9. Probability of licking on wrong trials, overtrained, Monkey B (left): P = 0.62; Mann-Whitney U test; Monkey S (right): P = 0.99; t-test, N=25.
10. Probability of licking on wrong trials, learning, Monkey B (left): P = 0.27; t-test; Monkey S (right): P = 0.80; paired t-test, N=25.
11. Visual exploration on wrong trials, overtrained, Monkey B: P = 0.59; Mann-Whitney U test; Monkey S: P = 0.60; Mann-Whitney U test, N=25.
12. Visual exploration on wrong trials, learning, Monkey B: P = 0.24; ranskum test; Monkey S: P = 0.10; paired t-test, N=25.

**
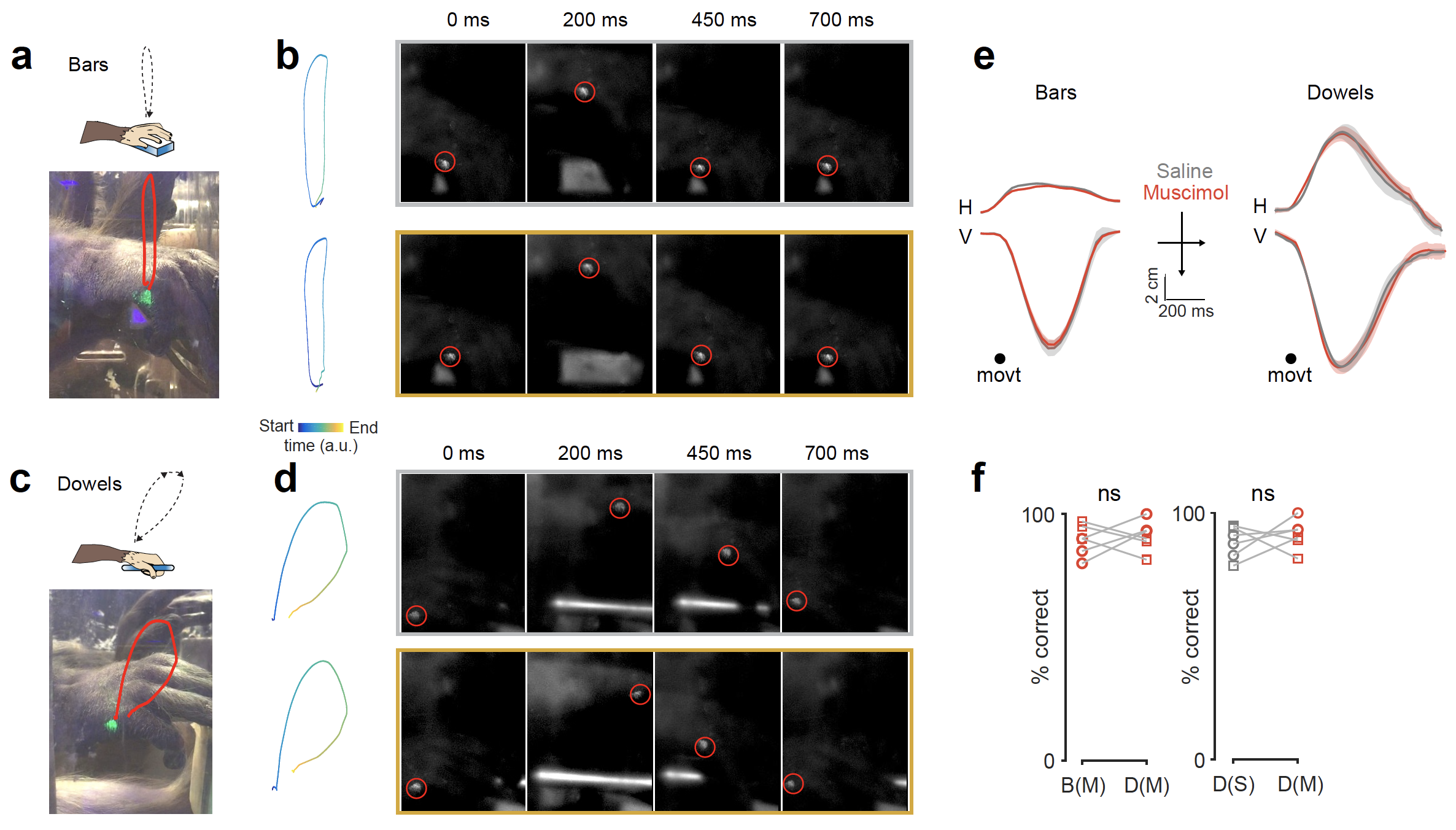
**

**Figure S8: Lateral-posterior cerebellar inactivation did not impair the ability to make different hand movements**

1. Top: Schematic of the bar release hand movement. Bottom: photo of the monkey’s hand holding a bar and the green fluorescent marker on the monkey’s hand. Red trace maps the position of the green fluorescent marker in space through the hand movement on an example trial.
2. Top left: Hand position in space and time (color indicates the time as in the color bar inset) for a representative trial during saline condition. Right: Snapshots from high-frame rate movies showing the monkey’s hand movement trajectory at four time points (0, 200 and 450 and 700 ms from the start of movement) for bar release. Red circle highlights the fluorescent marker. Bottom: same as top, but for a representative trial during Muscimol condition.
3. Same as **a**, but for dowel release condition.
4. Same as **b**, but for dowel release condition.
5. Left: Average horizontal (H) and vertical (V) hand trajectories for bar release, aligned on movement onset during saline (grey) and Muscimol (red) conditions. Right: Same as left but for dowels condition. Data is shown as mean ± SEM.
6. Behavioral performance of monkeys (monkey B: circles, monkey S: squares) during overtrained task for bars vs dowels under Muscimol (left; P = 0.37; paired t-test, N=6); and between dowels for saline and Muscimol (right; P = 0.70; paired t-test, N=6)

.

**Figure S9: Inactivation of the anterior cerebellum did not affect the monkey’s ability to learn new visuomotor associations**

1. For each monkey (B, top and S, bottom), the left panel shows a T1-weighted MRI image of the cerebellar hemisphere with muscimol infusion location (green outline) in the coronal plane. Lobules of interest are outlined in different colors. The right panel shows a flattened map reconstruction of the cerebellar cortex surface, with the infusion location marked in teal. Scale bar 5 mm. Cr, crus; D, dorsal; M, medial.
2. Behavioral performace of both monkeys during novel learning sessions for control-saline (gray) condition.
3. Behavioral performace of both monkeys during novel learning sessions for anterior cerebellar inactivation (green) condition.
4. Same analaysis as **Fig 1h** but for anterior cerebellum inactivation condition.
5. Same as **Fig 1i** but for anterior cerebellum inactivation condition.
6. Anterior cerebellar inactivation did not affect the strategy used by the monkey during.
7. Reaction time vs % error for saline injections (S; gray), for muscimol injections in the lateral-posterior cerebellum (M_ml_; red) and for lateral anterior cerebellum (M_a_; green). Each maker is an individual session (for one repetition of association learning). Triangles with error bars are means and s.e.m.
8. Left: The % error between saline (S) and muscimol in lateral-posterior cerebellum (M_ml_) was significant (***p<0.001; Mann-Whitney U test) and so was between muscimol in anterior cerebellum (M_a_) and muscimol in lateral-posterior cerebellum (M_ml_) (***p<0.001; Mann-Whitney U test) but not between S and M_a_ (0.12; Mann-Whitney U test). Right: The reaction time between S and M_ml_ was significant (***p<0.001; Mann-Whitney U test) and so was between M_a_ and M_ml_ (0.06; Mann-Whitney U test) but not between S and M_a_ (0.87; Mann-Whitney U test)

Data is shown as mean ± SEM.

**Figure S10: Muscimol injection sites**

For each monkey (B, left and S, right) top panels show muscimol injections outlines on T1-weighted MRI images in the coronal (left) and sagittal (right) planes. Lobules of interest are outlined in different colors. White arrows in the coronal panels indicate the level of the corresponding sagittal section.

Scale bar 5 mm. Cr, Crus; D, dorsal; M, medial.


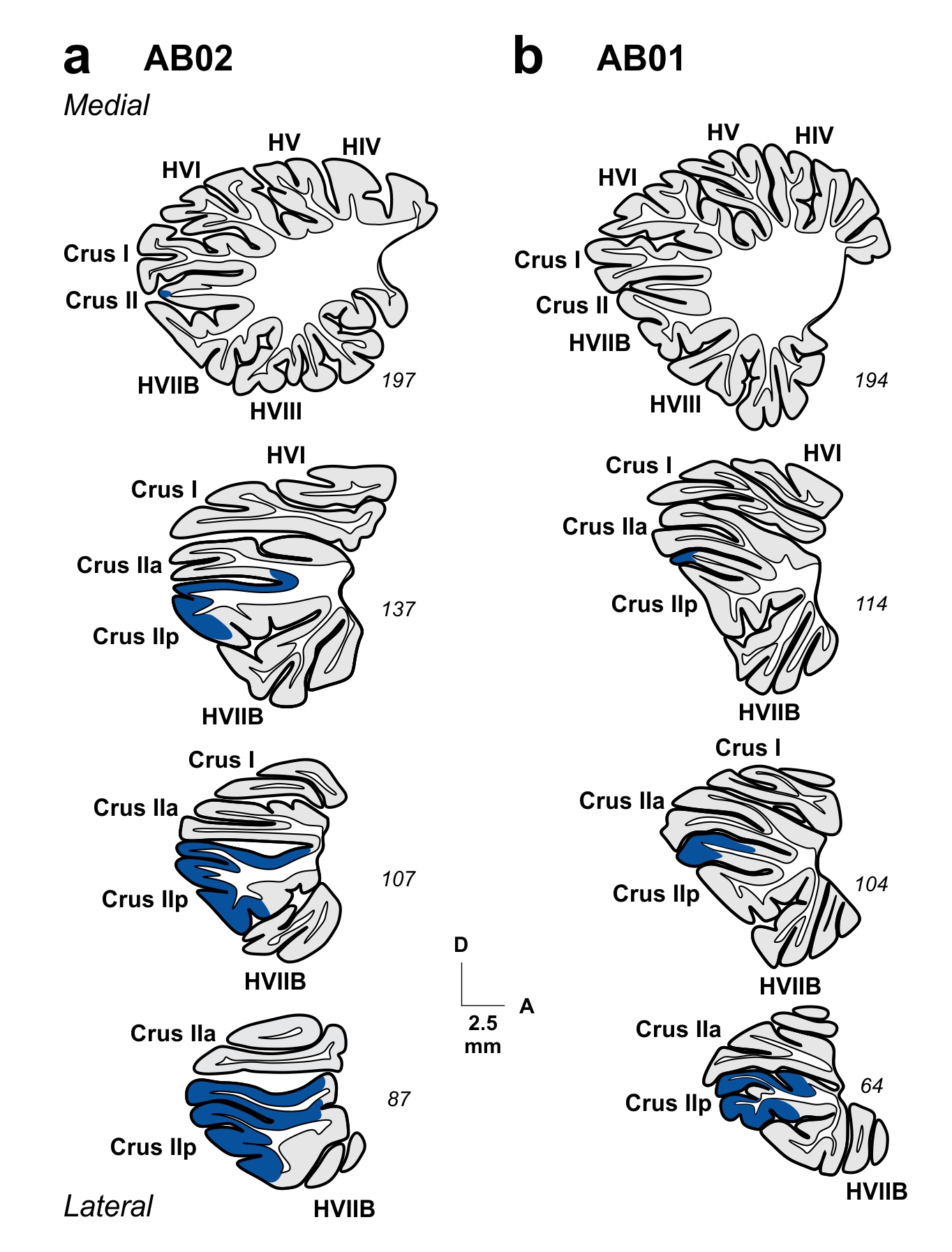


**Figure S11: Extent of virus injection sites in the cerebellar cortex.**

1. Drawings of representative sagittal sections through cerebellar cortex depict the extent of injection site in case AB02 (summary flat map depicted in **Fig 3c**, left panel). Sections are shown medial (top) to lateral (bottom) and correspond to tick marks in **Fig 3c**. Section numbers are in italics. Blue outlines indicate injection site outlines based on co-injected cholera toxin subunit b (CTb). A, anterior; D, dorsal.
2. As in **a** for case AB01 (summary map depicted in **Fig 3c**, right panel).


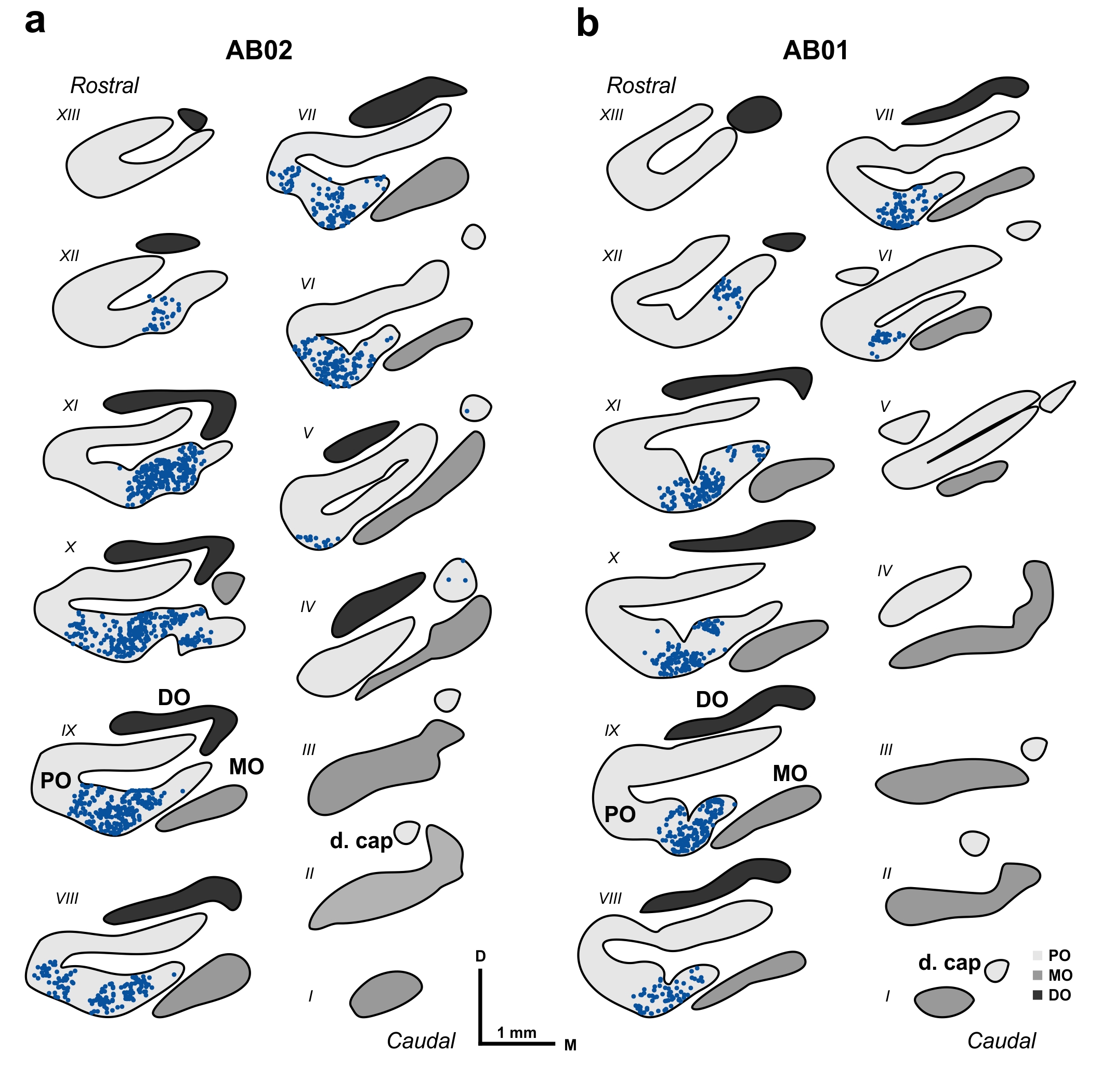


**Figure S12: Olivocerebellar projections to injection sites in Crus II.**

1. Drawings of 13 equally spaced (0.5 mm) 13 (I - XIII) transverse sections through the inferior olivary complex show locations of neurons labeled by retrograde transport of cholera toxin subunit beta (CTb) from injections into Crus IIp, case AB02 (injection site depicted in **Fig 3c**, left panel). Each blue dot represents one CTb labeled neuron. Sections are shown rostral (XIII) to caudal (I) in two columns. Light to dark gray shading identifies the: 1) principal olive (PO) and dorsal cap (d. cap), 2) medial accessory olive (MO) and attached groups, and 3) dorsal accessory olive (DO). D, dorsal; M, medial.
2. As in **a** for case AB01 (injection site depicted in **Fig 3c**, right panel).


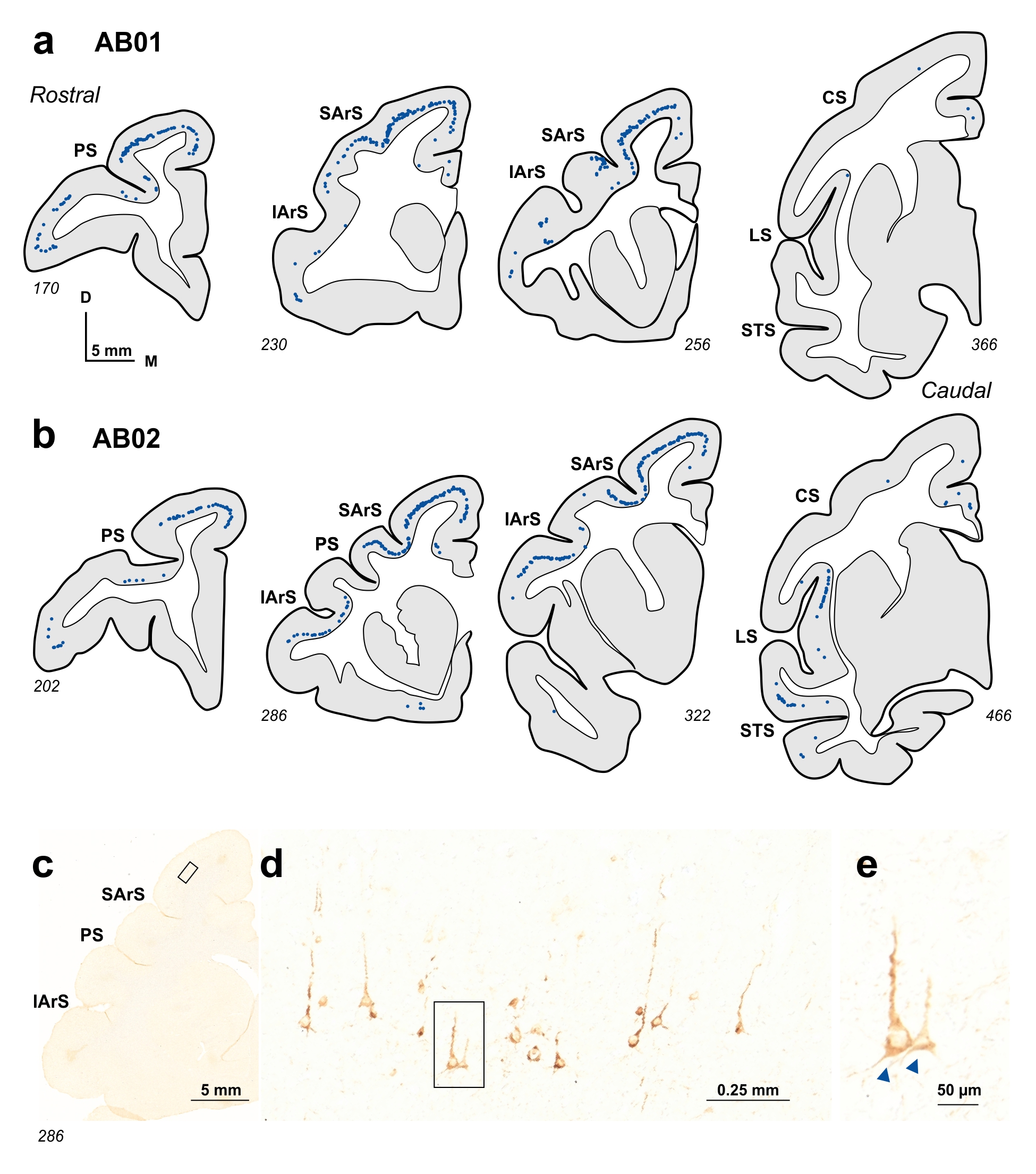


**Figure S13: Example second-order rabies virus labeling in the prefrontal cortex.**

1. Drawings of representative coronal sections through the cerebral cortex depict locations of rabies virus infected neurons in case AB01 (summary map in **Fig 3b**, left panel). Sections are shown anterior to posterior and correspond to tick marks in **Fig 3b**. Section numbers are in italics. Each blue dot represents one rabies virus infected neuron. CS, central sulcus; D, dorsal; IArS, inferior limb of the arcuate sulcus; IPS, intraparietal sulcus; LS, lateral sulcus; M, medial; PS principal sulcus; SArS, superior limb of the arcuate sulcus; STS, superior temporal sulcus.
2. As in **a** for case AB02 (summary map depicted in **Fig 3b**, right panel).
3. Photomicrograph of section 286 in AB02. Rectangle outlines the region that is magnified in **d**.
4. Example rabies virus infected neurons in the prefrontal cortex. Rectangle outlines the region that is magnified in **e**.
5. Magnification of area outlined in **d**. Blue arrowheads point to rabies infected neurons.


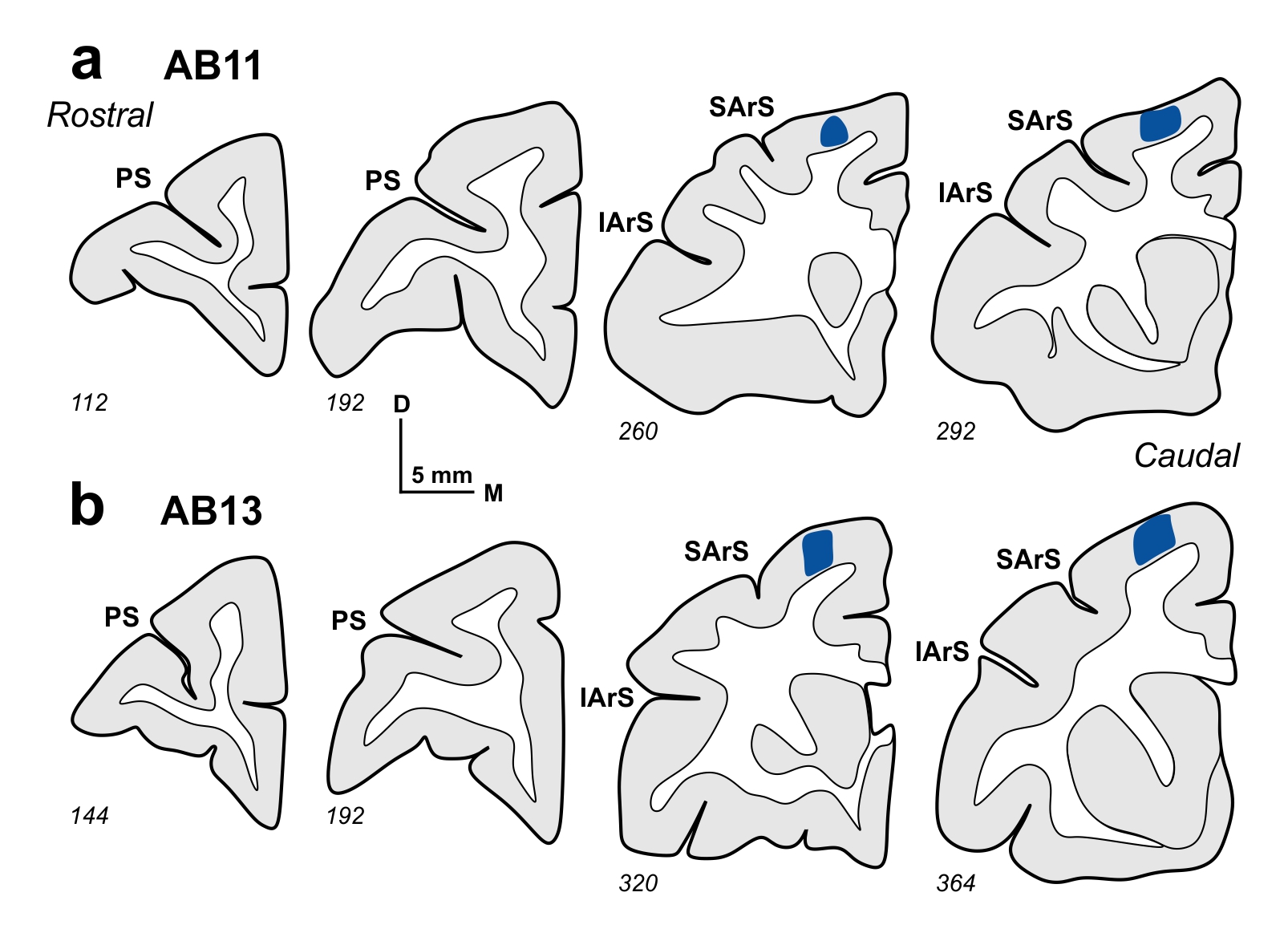


**Figure S14: Extent of virus injection sites in the prefrontal cortex.**

1. Drawings of representative coronal sections through the cerebral cortex depict the extent of injection site in case AB11 (summary map in **Fig 4b**, left panel). Sections are shown anterior to posterior and correspond to tick marks in **Fig 4c**. Section numbers are in italics. Blue outlines indicate injection site outlines based on co-injected cholera toxin subunit b (CTb). Abbreviations as in **Fig S13**.
2. As in **a** for case AB13 (summary map depicted in **Fig 4b**, right panel).


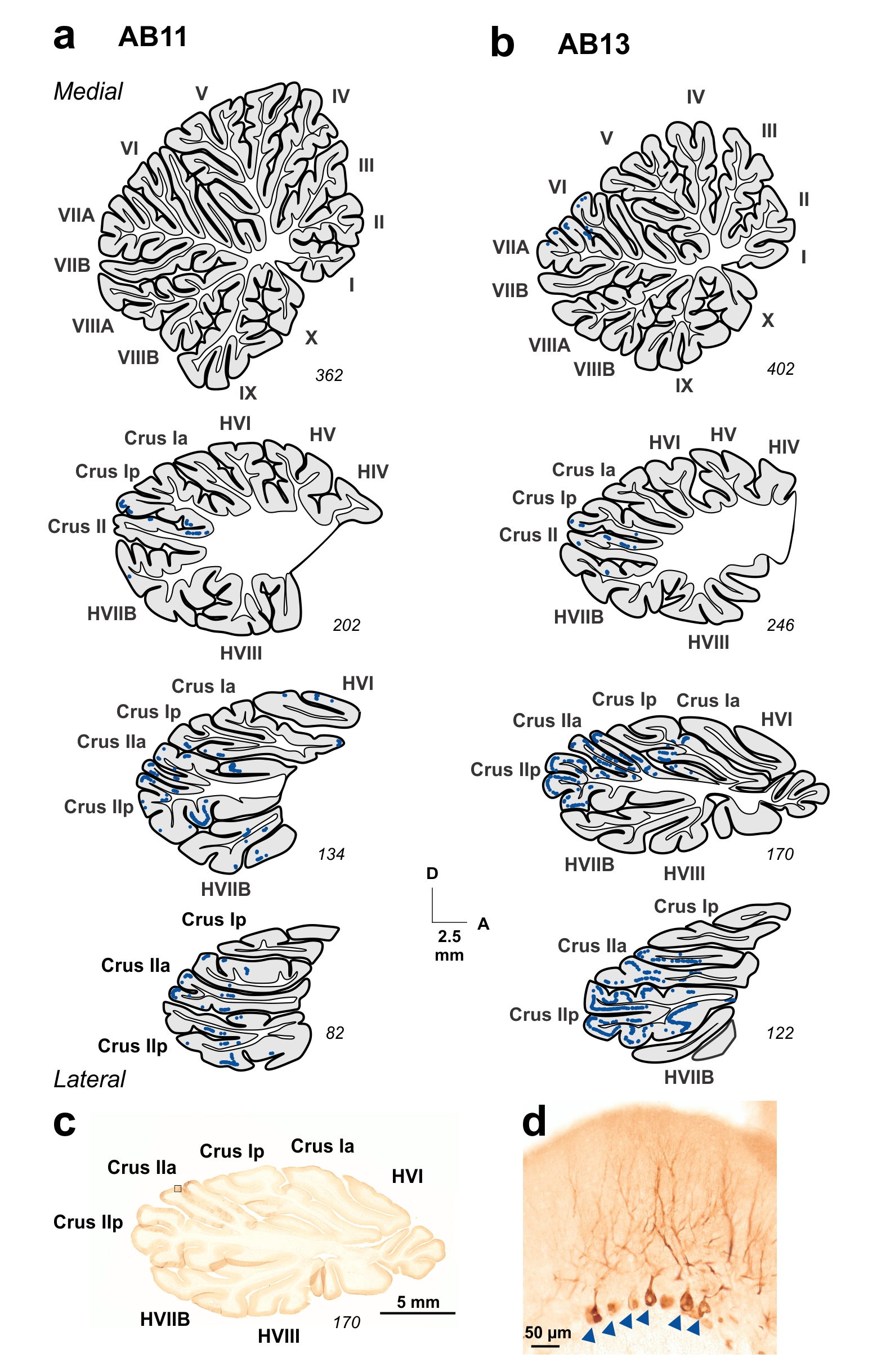


**Figure S15: Example third-order rabies virus labeling in the cerebellar cortex.**

1. Drawings of representative sagittal sections through the cerebellar cortex depict locations of rabies virus infected neurons in case AB11 (summary map in **Fig 4c**, left panel). Sections are shown medial to lateral and correspond to tick marks in **Fig 4c**. Section numbers are in italics. Each blue dot represents one rabies virus infected neuron. A, anterior; D, dorsal.
2. As in **a** for case AB13 (summary map depicted in **Fig 4c**, right panel).
3. Photomicrograph of section 170 in AB13. Rectangle outlines the region that is magnified in **d**.
4. Magnification of area outlined in **d**. Blue arrowheads point to rabies infected Purkinje cells.


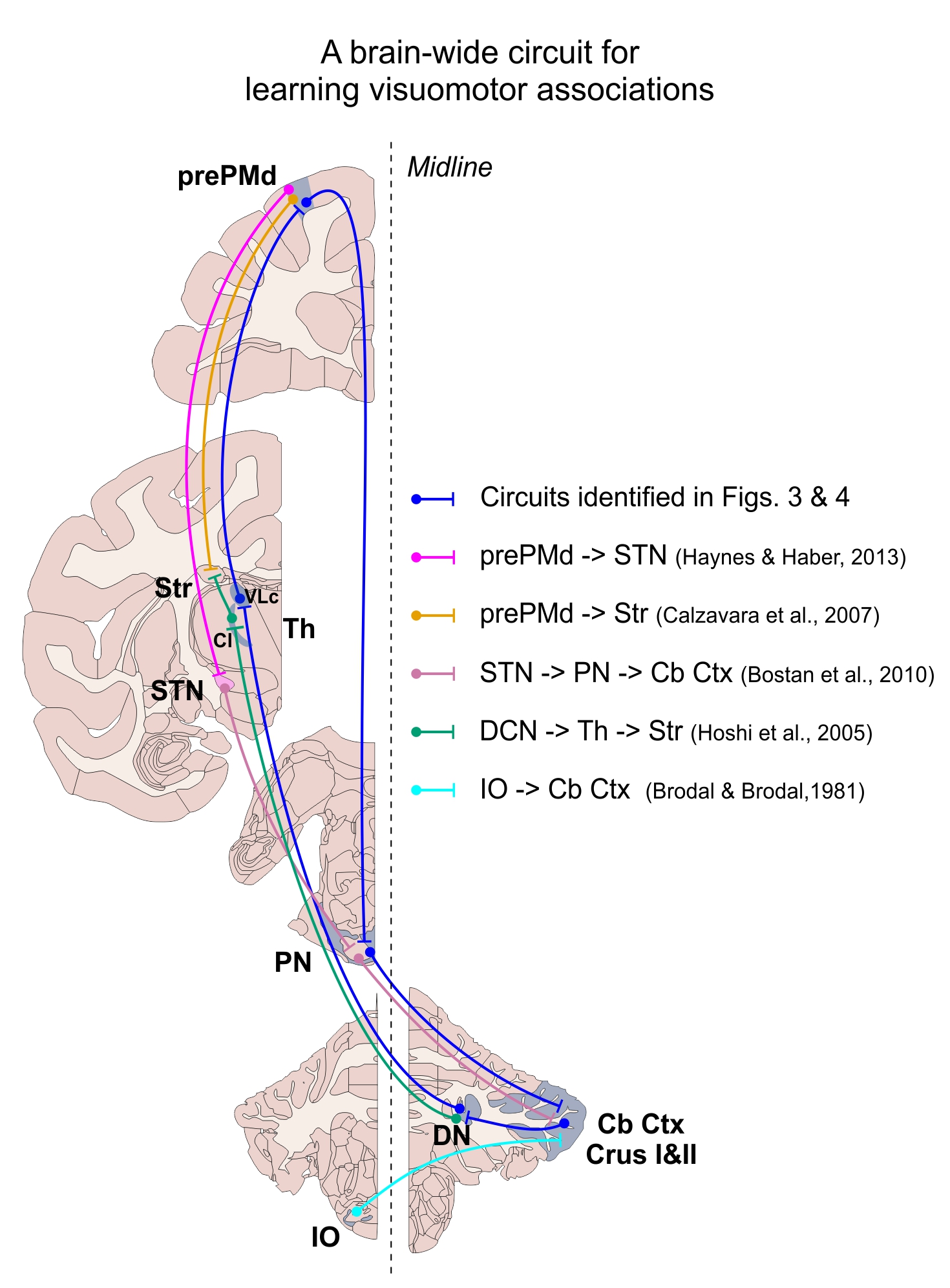


**Figure S16: An anatomical framework involving the cerebellum in a network for learning visuomotor association**

Summary diagram of the cerebro-cerebellar circuits for learning visuomotor associations and relevant anatomical connections in monkeys ^8-13^. Cb Ctx, cerebellar cortex; Cl, thalmic nucleus centralis lateralis; DN, dentate nucleus; IO, inferior olive; PN, pontine nuclei; prePMd, pre dorsolateral premotor cortex; STN, subthalamic nucleus; Str, striatum; Th, thalamus; VLc, thalamic nucleus ventralis lateralis pars caudalis.
