# Supplementary material for: A cerebro-cerebellar network for learning visuomotor associations
Source: Nat Commun. 2024 Mar 21;15:2519. doi: 10.1038/s41467-024-46281-0 (PMC10957870; doi:10.1038/s41467-024-46281-0)
Supplement: Supplementary file 2 — Reporting Summary [file 41467_2024_46281_MOESM2_ESM.pdf]

nature portfolio

Corresponding author(s): Naveen Sendhilnathan

Last updated by author(s): Jan 31, 2024

Reporting Summary

Nature Portfolio wishes to improve the reproducibility of the work that we publish. This form provides structure for consistency and transparency in reporting. For further information on Nature Portfolio policies, see our Editorial Policies and the Editorial Policy Checklist.

Statistics

For all statistical analyses, confirm that the following items are present in the figure legend, table legend, main text, or Methods section.

n/a	Confirmed	
☐	☒	The exact sample size (*n*) for each experimental group/condition, given as a discrete number and unit of measurement
☐	☒	A statement on whether measurements were taken from distinct samples or whether the same sample was measured repeatedly
☐	☒	The statistical test(s) used AND whether they are one- or two-sided<br>*Only common tests should be described solely by name; describe more complex techniques in the Methods section.*
☐	☒	A description of all covariates tested
☐	☒	A description of any assumptions or corrections, such as tests of normality and adjustment for multiple comparisons
☐	☒	A full description of the statistical parameters including central tendency (e.g. means) or other basic estimates (e.g. regression coefficient) AND variation (e.g. standard deviation) or associated estimates of uncertainty (e.g. confidence intervals)
☐	☒	For null hypothesis testing, the test statistic (e.g. *F*, *t*, *r*) with confidence intervals, effect sizes, degrees of freedom and *P* value noted<br>*Give P values as exact values whenever suitable.*
☒	☐	For Bayesian analysis, information on the choice of priors and Markov chain Monte Carlo settings
☒	☐	For hierarchical and complex designs, identification of the appropriate level for tests and full reporting of outcomes
☐	☒	Estimates of effect sizes (e.g. Cohen's *d*, Pearson's *r*), indicating how they were calculated

Our web collection on statistics for biologists contains articles on many of the points above.

Software and code

Policy information about availability of computer code

Data collection	ePhys - glass-coated tungsten electrodes with an impedance of 0.8-1.2 MOhms (FHC), Hitachi microdrive, FHC Neurocraft head stage, KrohnHite filter, Spike 2- Micro 1 401 system - CED electronics, NEI REX-VEX system. Video camera - Edmund Optics.<br>MD2 and MD3 (Accustage).
Data analysis	MATLAB (Mathworks version 2016b), Spike2 CED electronics version 8, Track Mate-Image J, ReconWin 2020 (Great Island Software).

For manuscripts utilizing custom algorithms or software that are central to the research but not yet described in published literature, software must be made available to editors and reviewers. We strongly encourage code deposition in a community repository (e.g. GitHub). See the Nature Portfolio guidelines for submitting code & software for further information.

Data

Policy information about availability of data

All manuscripts must include a data availability statement. This statement should provide the following information, where applicable:

- Accession codes, unique identifiers, or web links for publicly available datasets
- A description of any restrictions on data availability
- For clinical datasets or third party data, please ensure that the statement adheres to our policy

All the relevant data are that support the findings of this study are available at https://github.com/naveen-7/Cerebellum_reward. A reporting summary for this article is available as a Supplementary Information file. Source data are provided with this paper.

Research involving human participants, their data, or biological material

Policy information about studies with human participants or human data. See also policy information about sex, gender (identity/presentation), and sexual orientation and race, ethnicity and racism.

Reporting on sex and gender	N/A
Reporting on race, ethnicity, or other socially relevant groupings	N/A
Population characteristics	N/A
Recruitment	N/A
Ethics oversight	N/A

Note that full information on the approval of the study protocol must also be provided in the manuscript.

Field-specific reporting

Please select the one below that is the best fit for your research. If you are not sure, read the appropriate sections before making your selection.

☒ Life sciences ☐ Behavioural & social sciences ☐ Ecological, evolutionary & environmental sciences

For a reference copy of the document with all sections, see nature.com/documents/nr-reporting-summary-flat.pdf

Life sciences study design

All studies must disclose on these points even when the disclosure is negative.

Sample size	Sample size was not predetermined. We did not do a power analyses. We used similar numbers of monkeys and cells that are consistent with other studies of single unit activities in awake behaving monkeys (e.g., Medina et al. 2008; Yang et al. 2014) and virus tracing studies in nonhuman primates (e.g., Bostan et al. 2010).
Data exclusions	No data was excluded.
Replication	All attempts at replication were successful. We performed behavioral experiments and analyses in 2 monkeys. The results between these monkeys were comparable. Neuroanatomical tracing experiemnts for each of our injection sites (cerebellar cortex and prePMd) were performed (and replicated) in two monkeys.
Randomization	Randomization was not performed because both both behavioral monkeys participated in all the experimental conditions. The order of symbols appearing in the experiments was randomized so that the monkeys could not predict the upcoming stimulus identity.
Blinding	Blinding was not performed because both behavioral monkeys participated in all experimental conditions.

Reporting for specific materials, systems and methods

We require information from authors about some types of materials, experimental systems and methods used in many studies. Here, indicate whether each material, system or method listed is relevant to your study. If you are not sure if a list item applies to your research, read the appropriate section before selecting a response.

Materials & experimental systems

n/a	Involved in the study
☐	☒ Antibodies
☒	☐ Eukaryotic cell lines
☒	☐ Palaeontology and archaeology
☐	☒ Animals and other organisms
☒	☐ Clinical data
☒	☐ Dual use research of concern
☒	☐ Plants

Methods

n/a	Involved in the study
☒	☐ ChIP-seq
☒	☐ Flow cytometry
☒	☐ MRI-based neuroimaging

Antibodies

Antibodies used

We used a mouse monoclonal antibody (clone M957) that targets the rabies virus phosphoprotein (Rabies Centre of Experitse, Canadian Food Inspection Agency, Canada; dilution 1:300) to detect rabies virus. We use the goat anti-Cholera Toxin B Subunit (List Biological Laboratories, Campbell CA; Cat #703, RRID:AB_10013220; dilution 1:10,000) antibody to detect Subunit B of Cholera Toxin.

Validation

Monoclonal antibody (M957) for rabies virus has been generated and validated by the Rabies Centre of Expertise of the Canadian Food Inspection Agency (Nadin-Davis S.A. et al. 2000). Both M957 and the CTb antibody (#703 List Biological Laboratories) have been validated and are routinely used in non-human primate tissue (e.g. Bostan et al. 2010; Cerkevich et al. 2022; Coffman et al. 2011).

Animals and other research organisms

Policy information about studies involving animals; ARRIVE guidelines recommended for reporting animal research, and Sex and Gender in Research

Laboratory animals

Macaca mulatta (2 Male, 10 - 11 kg). Cebus apella (3 Female, 1 Male: AB1 F, 14 yo, 2.6 kg; AB2 M, 6 yo, 3.4 kg; AB11 F, 5 yo, 2.7 kg; AB13 F, 3 yo, 2.3 kg)

Wild animals

The study did not involve wild animals.

Reporting on sex

We used 3 male and 3 female monkeys for this study.

Field-collected samples

No field collected samples were used in this study.

Ethics oversight

All physiological, behavioral and inactivation experimental protocols were approved by the Animal Care and Use Committees at Columbia University and the New York State Psychiatric Institute and complied with the guidelines established by the Public Health Service Guide for the Care and Use of Laboratory Animals. All anatomical tracing procedures were conducted in accordance with the Association for Assessment and Accreditation of Laboratory Animal Care and the National Institutes of Health Guide for the Care and Use of Laboratory Animals. The experimental protocols werw approved by the Institutional Animal Care and Use Committee and the Biosafety Committee at the University of Pittsburgh.

Note that full information on the approval of the study protocol must also be provided in the manuscript.

Plants

Seed stocks

N/A

Novel plant genotypes

N/A

Authentication

N/A
